# Effect of physical exercise on cognitive function after chemotherapy in patients with breast cancer: a randomized controlled trial (PAM study)

**DOI:** 10.1186/s13058-022-01530-2

**Published:** 2022-05-26

**Authors:** E. W. Koevoets, S. B. Schagen, M. B. de Ruiter, M. I. Geerlings, L. Witlox, E. van der Wall, M. M. Stuiver, G. S. Sonke, M. J. Velthuis, J. J. Jobsen, M. B. E. Menke-Pluijmers, E. Göker, C. C. van der Pol, M. E. M. M. Bos, L. W. Tick, N. A. van Holsteijn, J. van der Palen, A. M. May, E. M. Monninkhof, PAM study group, Annebeth W. Haringhuizen, Wim A. van der Steeg, Dirkje W. Sommeijer, Frederiek Terheggen, Charlotte Blanken-Peeters, Harold Fliervoet, Margrethe S. Schlooz-Vries, Tanja G. Frakking, Marc W. A. van Tilburg, Corina Oldenhuis, Maartje F. Sier

**Affiliations:** 1 Julius Center for Health Sciences and Primary Care, University Medical Center Utrecht, Utrecht University, P.O. Box 85500, 3508 GA Utrecht, The Netherlands; 2 Division of Psychosocial Research and Epidemiology, Netherlands Cancer Institute, Amsterdam, The Netherlands; 3 Brain and Cognition Group, University of Amsterdam, Amsterdam, The Netherlands; 4 Department of Medical Oncology, University Medical Center Utrecht, Utrecht University, Utrecht, The Netherlands; 5 Center for Quality of Life, Netherlands Cancer Institute, Amsterdam, The Netherlands; 6 Center of Expertise Urban Vitality, Faculty of Health, University of Applied Sciences, Amsterdam, The Netherlands; 7 Department of Medical Oncology, Netherlands Cancer Institute, Amsterdam, The Netherlands; 8 Netherlands Comprehensive Cancer Organisation (IKNL), Utrecht, The Netherlands; 9 Medical School Twente, Medisch Spectrum Twente, Enschede, The Netherlands; 10 Breast Clinic, Albert Schweitzer Hospital, Dordrecht, The Netherlands; 11 Department of Medical Oncology, Alexander Monro Hospital, Bilthoven, The Netherlands; 12 Department of Surgery, Alrijne Ziekenhuis, Leiderdorp, The Netherlands; 13 Department of Medical Oncology, ErasmusMC Cancer Institute, Rotterdam, The Netherlands; 14 Department of Internal Medicine, Máxima Medisch Centrum, Eindhoven, The Netherlands; 15 Breast Cancer Center, Reinier de Graaf Hospital, Delft, The Netherlands; 16 Department of Research Methodology, Measurement, Universiteit Twente, Enschede, The Netherlands

**Keywords:** Breast cancer, Cancer-related cognitive impairment, Cognition, Cognitive complaints, Exercise training, Physical exercise, Aerobic exercise, Strength exercise, Physical fitness

## Abstract

**Background:**

Up to 60% of breast cancer patients treated with chemotherapy is confronted with cognitive problems, which can have a significant impact on daily activities and quality of life (QoL). We investigated whether exercise training improves cognition in chemotherapy-exposed breast cancer patients 2–4 years after diagnosis.

**Methods:**

Chemotherapy-exposed breast cancer patients, with both self-reported cognitive problems and lower than expected performance on neuropsychological tests, were randomized to an exercise or control group. The 6-month exercise intervention consisted of supervised aerobic and strength training (2 h/week), and Nordic/power walking (2 h/week). Our primary outcome was memory functioning (Hopkins Verbal Learning Test-Revised; HVLT-R). Secondary outcomes included online neuropsychological tests (Amsterdam Cognition Scan; ACS), self-reported cognition (MD Anderson Symptom Inventory for multiple myeloma; MDASI-MM), physical fitness (relative maximum oxygen uptake; VO_2peak_), fatigue (Multidimensional Fatigue Inventory), QoL (European Organisation for Research and Treatment of Cancer Quality of Life Questionnaire; EORTC QLQ C-30), depression (Patient Health Questionnaire-9, Hospital Anxiety and Depression Scale; HADS), and anxiety (HADS). HVLT-R total recall was analyzed with a Fisher exact test for clinically relevant improvement (≥ 5 words). Other outcomes were analyzed using multiple regression analyses adjusted for baseline and stratification factors.

**Results:**

We randomized 181 patients to the exercise (*n* = 91) or control group (*n* = 90). Two-third of the patients attended ≥ 80% of the exercise sessions, and physical fitness significantly improved compared to control patients (B VO_2peak_ 1.4 ml/min/kg, 95%CI:0.6;2.2). No difference in favor of the intervention group was seen on the primary outcome. Significant beneficial intervention effects were found for self-reported cognitive functioning [MDASI-MM severity (B-0.7, 95% CI − 1.2; − 0.1)], fatigue, QoL, and depression. A hypothesis-driven analysis in highly fatigued patients showed positive exercise effects on tested cognitive functioning [ACS Reaction Time (B-26.8, 95% CI − 52.9; − 0.6) and ACS Wordlist Learning (B4.4, 95% CI 0.5; 8.3)].

**Conclusions:**

A 6-month exercise intervention improved self-reported cognitive functioning, physical fitness, fatigue, QoL, and depression in chemotherapy-exposed breast cancer patients with cognitive problems. Tested cognitive functioning was not affected. However, subgroup analysis indicated a positive effect of exercise on tested cognitive functioning in highly fatigued patients.

*Trial Registration Netherlands Trial Registry*: Trial NL5924 (NTR6104). Registered 24 October 2016, https://www.trialregister.nl/trial/5924.

**Supplementary Information:**

The online version contains supplementary material available at 10.1186/s13058-022-01530-2.

## Introduction

The number of breast cancer survivors dealing with late effects of cancer and its treatment has increased in recent years, due to increasing incidence and survival [1]. Among these late effects are cognitive complaints, which are reported by a large number of breast cancer patients, particularly after chemotherapy [2, 3]. In up to 60% of patients, impaired neuropsychological test performance is found, including impaired learning and memory functioning, attention, processing speed, and executive functioning [4]. These cognitive problems are generally of mild to moderate severity [4], and even a moderate decline in cognitive functioning can have a significant impact on quality of life and daily activities [5, 6]. Moreover, differences in cognition between chemotherapy-treated patients and controls can be detected up to 20 years after treatment [7], emphasizing the need of interventions targeting these cognitive problems.

Exercise training might be an effective non-pharmacological intervention to reduce cognitive problems after (breast) cancer treatment [8]. Whereas many interventions, such as cognitive rehabilitation approaches, target the consequences of cognitive decline, exercise training might affect its underlying mechanisms. Rodent studies have shown that several biological processes affected by chemotherapy improved after exercise training, in particular hippocampal neurogenesis, which is important for learning and memory functioning [9]. Additionally, exercise interventions might also indirectly reduce cognitive problems by targeting fatigue, an important correlate of both exercise training and cancer-related cognitive problems [10].

Favorable effects of exercise training on cognition have been repeatedly observed in other populations, such as healthy elderly and patients with mild cognitive impairment [11, 12]. Recently, a large observational study showed positive effects of high physical activity levels before and during chemotherapy on cognitive functioning, even 6 months after chemotherapy treatment completion [13]. In breast cancer patients, only few small (n ranged between 17 and 87) exercise intervention studies have been performed after treatment. Although these studies provided preliminary support for positive effects of exercise programs on memory [14] and other cognitive functions [14–16], sufficiently powered trials with cognition as primary outcome measure are needed to establish or dismiss the role for exercise on cognition in cancer patients [17].

In the Physical Activity and Memory (PAM) study, we investigated the effects of a 6-month exercise intervention on cognitive functioning in cognitively impaired (self-reported and confirmed by tests) breast cancer patients who were diagnosed and treated with chemotherapy 2–4 years before study enrolment.

## Methods

### Design

The PAM study is a multi-center randomized clinical trial comparing a 6-month exercise intervention and a control group. Data were collected between December 2016 and September 2020. Measurements took place at the University Medical Center (UMC) Utrecht (The Netherlands) before randomization and after 6 months. The study was approved by the Medical Ethics Committee of the UMC Utrecht, and all patients provided written informed consent.

A detailed description of the PAM study design and recruitment has been published previously [18]. Additional measurements, such as neuroimaging, will be described separately.

### Patients

Female patients, 2–4 years after stage I–III breast cancer diagnosis, were eligible for inclusion if treated with (neo)adjuvant chemotherapy, between 30 and 75 years old, had no evidence of disease recurrence, reported ≤ 150 min of moderate-to-vigorous physical activity per week, had sufficient proficiency of the Dutch language, and were willing to be randomized. Moreover, patients needed to self-report cognitive problems after cancer diagnosis, which was confirmed by lower than expected performance on neuropsychological testing (see subsection ‘recruitment and randomization’). Exclusion criteria were: contraindication for exercise participation or MRI scanning, known neurological disorders that affect cognition (e.g., dementia, multiple sclerosis), and planned switches or stops of endocrine therapy < 4 months prior to the start or during the study period.

### Recruitment and randomization

**Fig. 1:**
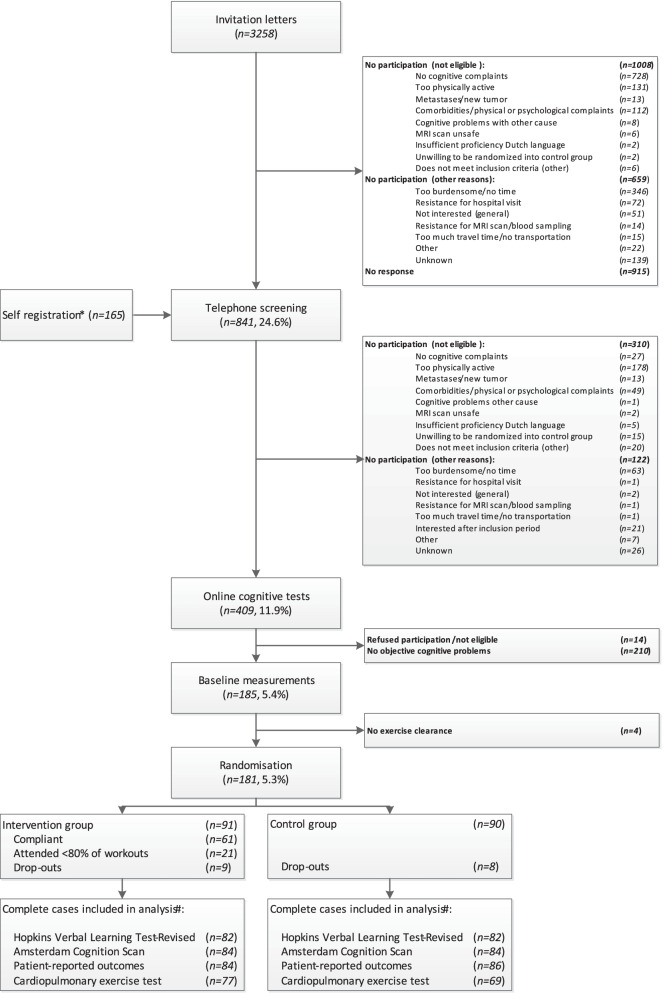
Flowchart of inclusion and randomization procedures of the Physical Activity and Memory (PAM) study patients. *Information through social media, pamphlets and by word of mouth. ^#^During the COVID-19 pandemic, seven patients completed the exercise program partly at home. The HVLT-R was assessed during video calls instead of face-to-face (*n* = 13). Less cardiopulmonary exercise tests were performed (missing*: n* = 13)

Patients were recruited through invitation letters (*n* = 3258) or self-registration (*n* = 165) (Fig. 1). After eligibility screening by phone (including a semi-structured interview about self-reported cognitive complaints), 409 (11.9%) patients completed the online Amsterdam Cognition Scan (ACS), to identify cognitive problems [19] (Table 1). ACS scores resulted in 11 outcomes in five different cognitive domains. Patients performing ≥ 1 normative standard deviation below average performance of healthy females aged 30–75 years, on at least two scores in different cognitive domains, were eligible for inclusion. This is an acceptable cutoff to define cognitive decline [20, 21]. After baseline measurements, patients were randomly allocated (1:1) by a member of the study team to the intervention or control group using a computer-generated sequence ensuring blinded treatment allocation provided by the data-management department (UMC Utrecht), stratified by age category (30–45, 45–60, 60–75 years) and endocrine therapy (yes, no).

**Table 1:** Content of the Amsterdam cognition scan

Test domain	Online test	Main outcome measures	Traditional equivalent
Learning and memory	Wordlist Learning Wordlist delayed recall and Wordlist Recognition	Total number of correct response (learning: trials 1 to 5)	Dutch version of Rey Auditory Verbal Learning Test (immediate recall, delayed recall and recognition)
Attention and working memory	Box tapping Digit sequences I Digit sequences II	Total number of correctly repeated sequences	Corsi block-tapping test WAIS-III digit span forward WAIS-III digit span backward
Processing speed	Reaction Time Connecting the dots I	Mean Reaction Time (ms) Completion time (s)	Visual Reaction Time (subtest FePsy) Trail making test A
Executive functioning	Connecting the dots II Place the Beads	Completion time (s) Total number of extra moves	Trail making test B Tower of London, Drexel University (ToL-dx)
Motor functioning	Fill the grid	Completion time (s)	Grooved pegboard

### Exercise intervention

The 6-month exercise intervention of 4 h/week included aerobic and strength training supervised by a physiotherapist close to the patients’ home (2 h/week) and Nordic/power walking (2 h/week), which could be carried out individually or in a group. Intensity of the program was tailored on women’s physical fitness level, based on baseline cardiopulmonary exercise testing, repetition maximum strength tests and potential constraints. Intensity of the supervised program increased as the program progressed, including high-intensity interval training starting in week 10 (Table 2). Approximately 1 month after the first training, the study team performed a monitoring visit to verify protocol adherence. Intensity of Nordic/power walking was set at 55–65% of the heart rate reserve (HRR); heart rate monitors were provided. Adherence to the supervised and walking sessions is registered in exercise logs by the patients and monitored by the physiotherapists.

**Table 2:** Supervised exercise program of the PAM study

Week	Aerobic	Strength
1–4	40–60% HRR	One circuit of 20–25 repetitions. Weights based on 20-RM tests (repeated every 4 weeks) Exercises: legs (squat, lunges, calve raises), arms (biceps curl, triceps extension), shoulder (shoulder press), thorax (Barbell bench press), back (rowing). Abdomen: crunch 30–40 repetitions
5–9	60–70% HRR 15–20 min, plus 70–89% HRR 5–10 min	
10–17	Interval training: 10 × 30 s vigorous to maximal exercise, alternated with 1 min active rest, plus 10 min 60–75% endurance	Two circuits of 15–20 repetitions. Weights based on 15-RM tests (repeated every 4 weeks) Exercises: legs (squat), arms (biceps curl, triceps extension), shoulder (shoulder press), thorax (Barbell bench press), back (rowing). Abdomen: crunch 30–40 repetitions; hoover/planking 2 × 45 s
18–26	Interval training: 2 circuits of 8 × 30 s vigorous to maximal exercise, alternated with 1 min active rest, plus 5 min 60–75% endurance	

*PAM* physical activity and memory, *HRR* heart rate reserve, *RM* repetition maximum

### Control group

Patients in the control group were requested to maintain their habitual physical activity level. A supervised 12-week exercise program was offered after study completion.

### Outcomes

Our primary outcome measure was the total recall score of the Hopkins Verbal Learning Test-Revised (HVLT-R) [22]. This face-to-face measure of memory functioning is the gold standard in neuro(oncological) trials and part of the core battery of the ICCTF [23]. A list of 12 words, from three semantic categories, was read three times to the patient. After each learning trial, the patient recalled as many words as possible. The sum of these learning trials comprised the total recall score (HVLT-R total recall). Parallel versions were used for baseline and follow-up measurements.

Other outcome measures for tested cognitive functioning included the HVLT-R delayed recall score and recognition trial (HVLT-R recognition), from which the recognition discrimination index was calculated (true-positives minus false-positives). Additionally, cognitive functioning was measured with an online test battery: the ACS [19], which was also used as a screening instrument for eligibility. All tests start with an instruction video and most tests have a practice session with feedback. The battery has shown 100% feasibility with only a few (resolvable) technical errors. Test–retest reliability was high (the total score had a test–retest reliability of 0.83), and concurrent validity was moderately-high to high. The ACS contains tests in the following cognitive domains: learning and memory, attention and working memory, processing speed, executive functioning, and motor functioning. The outcome measures including the corresponding test domains and traditional test equivalents are described in Table 1.

Self-reported cognitive functioning was measured with specific questions of the MD Anderson Symptom Inventory for multiple myeloma (MDASI-MM). Two questions on severity of memory and attention problems and their interference with daily functioning were included [24]. These questions are not disease specific and can be used in other populations.

Sociodemographic data (age and education), employment, menopausal status, and age at menopause were assessed by a self-developed questionnaire. Clinical characteristics were retrieved from medical records, and data on medication use (including endocrine therapy) were obtained during an interview.

For fatigue, five subscales of the Multidimensional Fatigue Inventory (MFI) were calculated (general fatigue, physical fatigue, mental fatigue, reduced motivation, and reduced activity) [25].

Quality of Life was measured with the European Organisation for Research and Treatment of Cancer Quality of Life Questionnaire (EORTC QLQ-C30). According to the manual, global health, and all functional scales including cognitive functioning, and three symptom scales (fatigue, pain, and insomnia) were calculated as well as a summary score, all ranging from 0 to 100, with higher scores representing better quality of life and functioning, and higher symptom burden [26, 27].

Symptoms of anxiety and depression were measured with the Hospital Anxiety and Depression Scale (HADS) [28]. Additionally, depression severity was measured with the total score of the Patient Health Questionnaire-9 (PHQ-9) [29].

Patients performed a maximal cycle cardiopulmonary exercise test using a ramp protocol with continuous breathing gas analysis and ECG monitoring. Relative maximum oxygen uptake [VO_2peak_] was calculated as an average over the final 30 s of exercise divided by body weight at baseline.

### Study adherence

Attendance was calculated separately for the supervised exercise program and Nordic/power walking and was defined as the number of attended sessions divided by the number of sessions offered. Compliance rate for the attended supervised sessions was calculated and averaged across all exercises by dividing the performed exercise volume by the prescribed volume.

### Statistical analysis

To detect a clinically relevant improvement in  ≥ 5 words (binary outcome) on the HVLT-R total recall [30–33] with 82% power and a meaningful difference (≥ 1 point) in self-reported complaints (MDASI-MM) with > 90% power (alpha = 0.05), a sample size of 90 patients per group was required, including a drop-out rate of 20%. First, for tested cognitive functioning impossible values and scores indicating computer/internet issues or poor understanding of test instructions (e.g., a score of 0 on Wordlist Learning) were removed from the database. Additionally, for tests where a higher score indicated worse performance (Reaction Time, Connecting the Dots I and II, Place the Beads and Fill the Grid), the (3.5×) median absolute distance (MAD) method was applied [34], for each age category (≤ 40, 41–59, and ≥ 60 years) separately, to prevent age-based bias. Moreover, the total Reaction Time score (mean of approximately 30 consecutive trials) was omitted if > 30% of the trials was removed due to outliers.

For primary analyses, the intention-to-treat principle was applied and based on complete case data [35]. Cognition analyses were repeated with multiple imputation (*n* = 10) for missing outcome data to prevent potential selection bias (Package: MICE [36]; R, 2017) [37]. Each patient’s HVLT-R total recall score recorded at 6 months was assigned a binary outcome as improvement or failure (stable or declined). Post-treatment improvement in the HVLT-R total recall score (≥ 5 words) in the intervention group was compared to the control group using Fisher exact test, and relative risks adjusted for stratification factors were calculated with a Poisson regression analysis.

All measures of tested cognitive functioning (three HVLT-R and eleven ACS measures), self-reported cognitive functioning (MDASI-MM severity and interference), physical fitness (VO_2peak_), and patient-reported outcomes (MFI, EORTC QLQ-C30, PHQ-9, and HADS) were analyzed with multiple regression analyses adjusted for stratification factors and baseline scores, to assess between-group differences.

As per protocol analyses, all analyses on cognitive outcomes were repeated for patients with a minimal attendance of 80%.

Pre-specified subgroup analyses for cognitive outcomes were performed for endocrine therapy (yes, no), age category (30–45, 45–60, 60–75 years), and menopausal status (pre-/peri-menopausal, postmenopausal). In addition, a post-hoc analysis for recently established clinically important fatigue scores on the EORTC QLQ-C30 fatigue scale (≥ 39) was performed [38].

Critical alpha value was set at 0.05 two-sided for all analyses. Analyses (except multiple imputation) were performed with IBM SPSS Statistics for Windows version 25.0.0.2 [39].

## Results

### Patient characteristics

We randomized 181 patients to the intervention (*n* = 91) or control group (*n* = 90). Patients were treated in 28 Dutch hospitals, aged 52 years on average, and educational level was middle to high. Baseline characteristics were comparable between groups, except for psychotropic medication (Table 3). Twenty patients, equally divided over the study groups, had an unplanned switch or stop of their endocrine therapy between eligibility screening and follow-up measurements. No serious adverse events were reported.

**Table 3:** Baseline demographic and treatment characteristics for the study groups of the PAM study

	Intervention group (*n* = 91)	Control group (*n* = 90)
Age (years)	52.1 (8.6)	52.5 (8.7)
Education level (*n* (%))		
High	42 (46.2)	36 (40.0)
Middle	49 (53.8)	52 (57.8)
Low	0 (0)	0 (0)
Missing	0 (0)	2 (2.2)
Employment (n (%))		
Yes (full/part-time)	51 (56.0)	47 (52.2)
Temporarily work disabled	6 (6.6)	8(8.9)
< 100%	3 (3.3)	1 (1.1)
100%	3 (3.3)	7 (7.8)
Work disabled	14 (15.4)	15 (16.7)
< 35%	0 (0)	0 (0)
35–80%	0 (0)	5 (5.6)
> 80%	14 (15.4)	10 (11.1)
No	17 (18.7)	18 (20)
Missing	3 (3.3)	2 (2.2)
Physical fitness (VO_2peak_ in ml/min/kg)	23.6 (4.7)	24.9 (6.2)
Menopausal status (*n* (%))		
Pre/peri	10 (11.0)	11 (12.2)
Post	81 (89.0)	79 (87.8)
Age of menopause (years)	47.4 (6.4)	47.0 (5.5)
Time since diagnosis (years)^a^	3.1 (0.7)	3.1 (0.6)
Tumor grade (*n* (%))		
I	11 (12.1)	8 (8.9)
II	36 (39.5)	38 (42.2)
III	31 (34.1)	34 (37.7)
Unknown	13 (14.3)	10 (11.2)
Surgery (*n* (%))^a^		
Yes	91 (100)	89 (98.9)
Unknown		1 (1.1)
Chemotherapy timing (*n* (%))		
Neoadjuvant	43 (47.3)	43 (47.8)
Adjuvant	44 (48.3)	42 (46.7)
Both	3 (3.3)	3 (3.3)
Unknown	1 (1.1)	2 (2.2)
Time since completion chemotherapy (years)^a^	2.6 (0.7)	2.6 (0.6)
Radiation (*n* (%))		
Yes	71 (78.0)	65 (72.2)
No	20 (22.0)	24 (26.7)
Unknown		1 (1.1)
Targeted therapy (*n* (%))		
Yes	19 (20.9)	19 (21.1)
No	72 (79.1)	69 (76.7)
Unknown		2 (2.2)
Endocrine therapy (*n* (%))		
Yes	57 (62.6)	54 (60.0)
No	34 (37.4)	36 (40.0)
Medication use (*n* (%))		
Cardiovascular	18 (19.8)	19 (21.1)
Anti-diabetic	2 (2.2)	2 (2.2)
Psychotropic	30 (33.0)	17 (18.9)
Pain medication	13 (14.3)	15 (16.7)

Values indicate mean (SD) unless indicated otherwise

^a^For time since diagnosis, average years were based on 83 intervention patients and 84 control patients. For time since completion chemotherapy, average years were based on 85 intervention patients and 79 control patients

Follow-up data for our primary outcome measure (HVLT-R total recall) were obtained from 82 patients in both the intervention and control group (attrition rate = 9.4%). Reasons for drop-out were: (possible) metastases/new (benign) tumor (*n* = 5), personal circumstances (*n* = 5), medical reasons (*n* = 3), or other (*n* = 4). Drop-outs were lower educated and used more often anti-diabetic and psychotropic medication.

Two-third of the intervention patients attended ≥ 80% of all exercise sessions (exercise supervised by physiotherapist: 69%; Nordic/power walking: 65%), with a median attendance of 88% (range 0–100%, mean = 75% ± 28). Moreover, patients had a median attendance of 46 sessions of the 52 offered supervised sessions. Median compliance to the attended supervised exercise sessions was 95% (range 71–100%, mean = 93% ± 7). This was reflected in a significant increase from baseline to follow-up in physical fitness in the intervention compared to the control group (B VO_2peak_ 1.39 ml/min/kg, 95%CI: 0.59; 2.19, ES = 0.26).

### Cognitive functioning

**Fig. 2:**
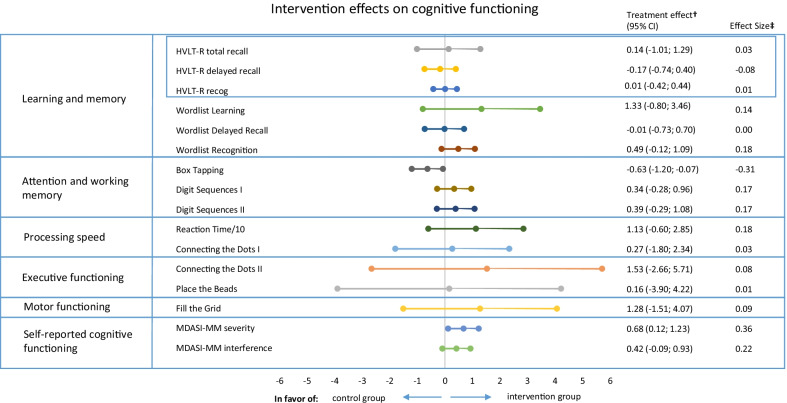
Exercise intervention effects on cognitive functioning in breast cancer patients. HVLT-R, Hopkins Verbal Learning Test-Revised; MDASI-MM, MD Anderson Symptom Inventory for multiple myeloma. Reaction Time in ms is divided by 10. ^†^The treatment effect is the regression coefficient of a linear regression analysis adjusted for baseline cognitive test score, age, and endocrine therapy. Tests/questionnaires for which a higher score indicated worse performance/more symptoms were inverted [Reaction Time, Connecting the Dots (I & II), Place the Beads, Fill the Grid, and MDASI-MM (Severity and Interference)]. Therefore, a positive score indicates a beneficial effect of the intervention. ^‡^Effect Sizes (ES) were calculated by dividing Beta by the pooled SD at baseline, with positive ESs meaning a beneficial effect of the intervention on a specific outcome. ESs < 0.2 indicate “no difference,” ESs between 0.2 and 0.5 indicate “small differences,” ESs between 0.5 and 0.8 indicate “medium differences,” and ESs ≥ 0.8 indicate “large differences” [50]. An ES of 0.5 or higher was considered clinically relevant [51]

We did not find a significant difference in the proportion of patients with an improvement on the HVLT-R total recall score between the intervention (11.0%) and control group (9.8%); RR = 1.11 (95%CI: 0.43; 2.87). Additionally, no between-group differences were found for other HVLT-R measures and ten of the eleven measures of the ACS (Fig. 2 and Additional file 1: Table S1). Box tapping showed significant differences at follow-up, in favor of the control group (B-0.63, 95% CI − 1.20; − 0.07). Self-reported cognitive functioning showed improvements in favor of the intervention group on the MDASI-MM severity scale (B-0.68, 95% CI − 1.23; − 0.12).

For tested and self-reported cognitive measures, between 11 and 20 values were missing at follow-up. Multiple imputation resulted in insignificant differences between groups for box tapping but did not change results of the remaining tested and self-reported cognitive outcomes.

Since a relevant difference at baseline was seen for psychotropic medication between groups, cognition analyses were repeated with this variable as covariate. This did not affect results significantly.

In the per protocol analyses, including patients with ≥ 80% exercise adherence, most effect sizes of tested cognitive outcome measures remained the same or slightly increased in favor of the intervention group (12/14 of the (online) cognitive outcomes). However, effect sizes remained small, and no additional significant effects were found (Additional file 1: Table S2).

Stratified analyses for endocrine therapy (yes/no) showed positive exercise effects on Digit Sequences I (B0.85, 95% CI 0.08; 1.63, ES = 0.39) and self-reported cognitive functioning (severity: B-0.72, 95% CI − 1.45; 0.004, ES = 0.37; interference: B-0.68, 95% CI − 1.34; − 0.01, ES = 0.35) only in patients using endocrine therapy. For patients without endocrine therapy, box tapping showed significant differences at follow-up, in favor of the control group (B-0.92, 95% CI − 1.69; − 0.16, ES = − 0.46). Stratified analyses for age category did not show consistent results in favor of the exercise or control group. Results did not differ clearly by menopausal status. See Additional file 1 for more detailed results of the stratified analyses (Additional file 1: Tables S3–S5).

**Fig. 3:**
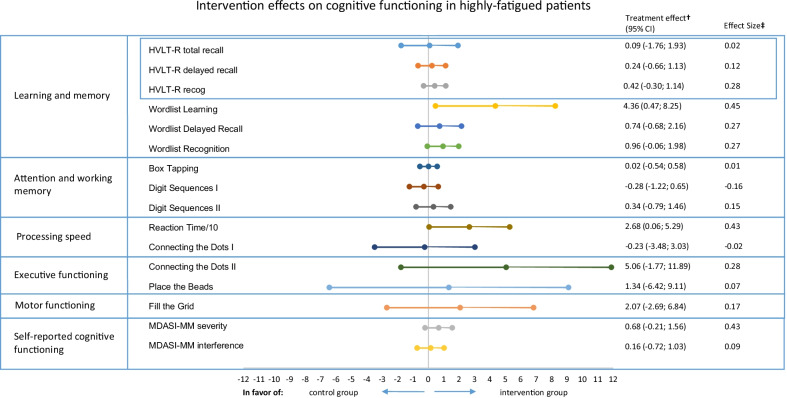
Exercise intervention effects on cognitive functioning in highly fatigued breast cancer patients. HVLT-R, Hopkins Verbal Learning Test-Revised; MDASI-MM, MD Anderson Symptom Inventory for multiple myeloma. Reaction Time in ms is divided by 10. ^†^The treatment effect is the regression coefficient of a linear regression analysis adjusted for baseline cognitive test score, age, and endocrine therapy. Tests/questionnaires for which a higher score indicated worse performance/more symptoms were inverted [Reaction Time, Connecting the Dots (I and II), Place the Beads, Fill the Grid, and MDASI-MM (Severity and Interference)]. Therefore, a positive score indicates a beneficial effect of the intervention. ^‡^Effect Sizes (ES) were calculated by dividing Beta by the pooled SD at baseline, with positive ESs meaning a beneficial effect of the intervention on a specific outcome. ESs < 0.2 indicate “no difference,” ESs between 0.2 and 0.5 indicate “small differences,” ESs between 0.5 and 0.8 indicate “medium differences,” and ESs ≥ 0.8 indicate “large differences” [50]. An ES of 0.5 or higher was considered clinically relevant [51]

Post hoc analyses for level of fatigue showed beneficial effects of exercise for the highly fatigued patients, on Wordlist Learning (B4.36, 95% CI 0.47; 8.25) and Reaction Time (B-26.7, 95% CI − 52.9; − 0.6), and a borderline significant improvement on Wordlist Recognition (B0.96, 95% CI − 0.06; 1.98) (Fig. 3 and Additional file 1: Table S6). Results on other cognitive outcomes are comparable to the intention-to-treat analysis.

### Patient-reported outcomes

**Fig. 4:**
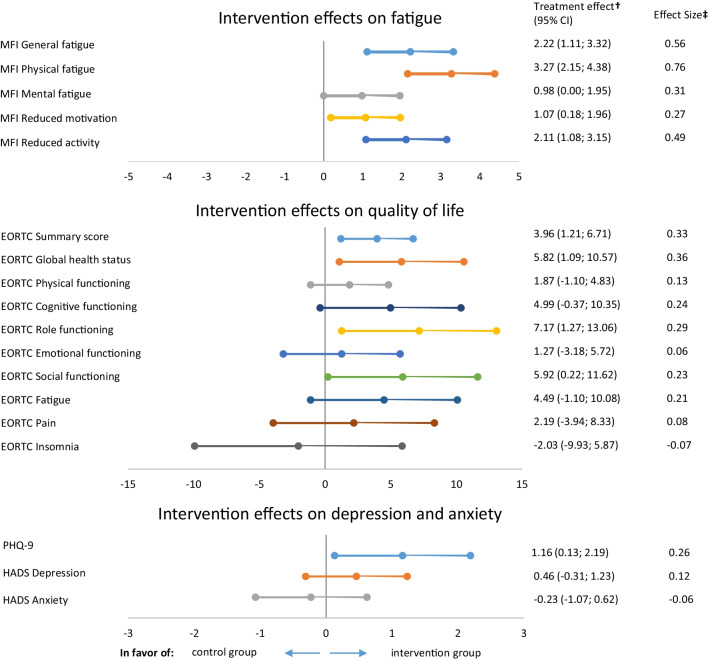
Exercise intervention effects on patient-reported outcomes. *MFI* Multidimensional Fatigue Inventory, *EORTC* European Organisation for Research and Treatment of Cancer Quality of Life Questionnaire, *PHQ-9* Patient Health Questionnaire-9, *HADS* hospital anxiety and depression scale. ^†^The treatment effect is the regression coefficient of a linear regression analysis adjusted for baseline scores, age, and endocrine therapy. Questionnaires for which a higher score indicated worse functioning/more symptoms were inverted (MFI subscales, EORTC Fatigue, EORTC Pain, EORTC Insomnia, PHQ-9, and HADS Depression and Anxiety). Therefore, a positive score indicates a beneficial effect of the intervention. ^‡^Effect Sizes (ES) were calculated by dividing Beta by the pooled SD at baseline, with positive ESs meaning a beneficial effect of the intervention on a specific outcome. ESs < 0.2 indicate “no difference,” ESs between 0.2 and 0.5 indicate “small differences,” ESs between 0.5 and 0.8 indicate “medium differences,” and ESs ≥ 0.8 indicate “large differences” [50]. An ES of 0.5 or higher was considered clinically relevant [51]

For fatigue (MFI), statistically significant between group differences at follow-up were observed favoring the intervention group [general fatigue (B-2.22, 95% CI − 3.32; − 1.11), physical fatigue (B-3.27, 95% CI − 4.38; − 2.15)], mental fatigue (B-0.98, 95% CI − 1.95; 0.00), reduced motivation (B-1.07, 95% CI − 1.96; − 0.18), and reduced activity (B-2.11, 95% CI − 3.15; − 1.08) (Fig. 4 and Additional file 1: Table S7).

For quality of life, significant beneficial effects of the exercise intervention were found for the EORTC QLQ C-30 summary score (B3.96, 95% CI 1.12;6.75), global health status (B5.82, 95% CI 1.09; 10.57), role functioning (B7.17, 95% CI 1.27; 13.06), social functioning (B5.95, 95% CI 0.22; 11.62), and with borderline significance on the cognitive functioning scale (B4.99, 95% CI − 0.37; 10.35) (Fig. 4 and Additional file 1: Table S7).

Depression severity (PHQ-9) significantly improved in the intervention group, compared to controls (B-1.16, 95% CI − 2.16; − 0.13) (Fig. 4 and Additional file 1: Table S7). For anxiety (HADS), no between group differences were observed (Fig. 4 and Additional file 1: Table S7).

## Discussion

The PAM study is the first sufficiently powered randomized controlled trial investigating the effects of a 6-month physical exercise intervention on cognitive function in patients with breast cancer who still had cognitive complaints and problems 2.5 years after completion of treatment with chemotherapy. The exercise intervention did not affect tested cognitive function in the total population. Interestingly, a hypothesis-driven analyses indicated a beneficial effect of exercise on tested cognition in highly fatigued patients. Moreover, in the total population, significantly positive intervention effects were seen for self-reported cognitive functioning, as well as physical fitness, fatigue, QoL, and depression.

These results are consistent with the roughly comparable study of Hartman et al. [16], which is the only exercise study with a sample size of > 10 patients per group (exercise group: *n* = 43) and with cognitive functioning as primary outcome. They did not find effects of exercise training on tested cognitive functioning in the total group and in patients > 2 years post-surgery, but an indication for a positive effect on self-reported cognitive functioning was found. Self-reported cognitive functioning is often related to psychosocial factors [40]. In the PAM study, we found, besides effects on self-reported cognitive functioning, favorable effects on fatigue, QoL, and depression. These exercise effects on psychosocial factors have previously been established in breast cancer patients [41].

Cognitive problems in cancer patients are multifactorially determined and various mechanisms exist by which cancer and cancer therapies give rise to both self-reported cognitive complaints and cognitive decline formally assessed by neuropsychological testing [42]. Understanding the underlying causes of cognitive problems is a prerequisite to develop and select the most beneficial clinical interventions. Our exercise intervention aimed at improving tested cognitive problems in patients who self-report cognitive complaints, by targeting one of the presumed causes of these problems, i.e., impaired hippocampal neurogenesis [9]. Although current results did not indicate statistically significant effects of exercise on memory function or other cognitive functions, 11/14 cognitive measures showed changes in favor of the intervention group. Effect sizes, moreover, increased by increasing exercise attendance. This suggests potentially much smaller effects of exercise on tested cognitive functioning than we anticipated, and only a larger trial could have detected these smaller effects. It is unclear whether these small effects would be of sufficient clinical relevance.

In a hypothesis-driven post hoc analysis, we found that patients who reported considerable symptoms of fatigue at baseline improved on tested cognitive functioning, in particular on tasks measuring learning and memory. Using a structural equation modeling framework, Ehlers et al. [10] studied pathways from physical activity to fatigue to cognitive performance. They concluded that effects of exercise on cognitive performance may be partially explained by the influence of exercise on cancer-related symptoms, including fatigue. Both fatigue and cancer-related cognitive impairment have been associated with (neuro)inflammation, one of the adverse effects of cancer (treatment) [4, 43]. Since physical activity might positively affect inflammatory status [44, 45], beneficial effects of exercise on cognitive functioning in chemotherapy-exposed cancer patients are probably not exclusively driven by targeting neurogenesis in the hippocampus. Additionally, these observations may advocate to select highly fatigued patients for enrolment in future exercise intervention studies aiming at improving cognition. This would be justifiable also because these patients are expected to benefit most from an exercise intervention with respect to fatigue outcomes [46].

A potential limitation of our study was that patients needed to improve their baseline HVLT-R Total Recall score, our primary outcome measure, by 5 words. Patients performed considerably better at this test at baseline than anticipated, requiring in some cases a near perfect performance at follow-up to show clinically relevant improvement. However, analyzing the HVLT-R and ACS Wordlist as continuous outcomes and defining less words as clinically relevant improvement did not show significant intervention effects as well (data not shown). Furthermore, we did not adjust for multiple testing in our secondary analyses; hence, false-positive findings cannot be excluded. For instance, our significant results on box tapping in favor of the control group, which were not in line with the general pattern of study results and absent after multiple imputation, were probably a false-positive finding.

Strengths of the study include our large study sample of cognitively impaired and inactive patients, and our long and intense (partly) supervised exercise training, including high intensity interval training [47, 48]. Additionally, our patients showed good adherence to the exercise program and physical fitness improved following the exercise intervention.

## Conclusion

Our research underscores the importance of careful evaluation of promising interventions using randomized controlled trials. Behavioral interventions, such as compensatory interventions, can improve self-reported cognitive complaints, but few have the capacity to actually improve cognitive test performance [49]. Our trial showed no overall benefit of physical exercise on tested cognitive functioning in chemotherapy-exposed breast cancer patients with cognitive problems and emphasizes the complexity surrounding physical exercise as a potent intervention. Physical exercise led to improved self-reported cognitive functioning, physical fitness, fatigue, QoL, and depression. The finding that physical exercise improved tested cognitive function in highly fatigued patients is a hopeful new avenue of research. Future research should focus on uncovering which patients benefit most from physical exercise interventions and investigate whether fatigue mediates or moderates the effect on cognitive performance.

## Supplementary Information

**Additional file 1**.** Table S1**. Intervention effects on cognitive functioning.** Table S2**. Intervention effects on cognitive functioning, per protocol.** Table S3**. Intervention effects, separately for patients with and without endocrine therapy.** Table S4**. Intervention effects, separately for patients of different age categories (30-44, 45-59, 60-75 years).** Table S5**. Intervention effects, separately for patients of with pre- and peri- menopausal status and patients with postmenopausal status.** Table S6**. Intervention effects on cognitive functioning, stratified for low versus high levels of fatigue measured with the EORTC QLQ C-30 fatigue scale.** Table S7**. Intervention effects on patient-reported outcomes.

## Data Availability

The datasets used during the current study are available from the corresponding author on reasonable request.

## Abbreviations

ACS: Amsterdam Cognition Scan
ECG: Electrocardiography
EORTC QLQ C-30: European Organisation for Research and Treatment of Cancer Quality of Life Questionnaire
HADS: Hospital Anxiety and Depression Scale
HVLT-R: Hopkins Verbal Learning Test-Revised
ICCTF: International Cognition and Cancer Task Force
MAD: Median absolute distance
MDASI-MM: MD Anderson Symptom Inventory for multiple myeloma
MFI: Multidimensional Fatigue Inventory
MRI: Magnetic resonance imaging
PAM study: Physical Activity and Memory study
PHQ-9: Patient Health Questionnaire-9
QoL: Quality of life
UMC: University Medical Center
VO_2peak_: Relative maximum oxygen uptake
