# Supplementary material for: Effect of physical exercise on cognitive function after chemotherapy in patients with breast cancer: a randomized controlled trial (PAM study)
Source: Breast Cancer Res. 2022 May 26;24:36. doi: 10.1186/s13058-022-01530-2 (PMC9135390; doi:10.1186/s13058-022-01530-2)
Supplement: Supplementary file 1 — Additional file 1. Table S1. Intervention effects on cognitive functioning. Table S2. Intervention effects on cognitive functioning, per protocol. Table S3. Intervention effects, separately for patients with and without endocrine therapy. Table S4. Intervention effects, separately for patients of different age categories (30-44, 45-59, 60-75 years). Table S5. Intervention effects, separately for patients of with pre- and peri- menopausal status and patients with postmenopausal status. Table S6. Intervention effects on cognitive functioning, stratified for low versus high levels of fatigue measured with the EORTC QLQ C-30 fatigue scale. Table S7. Intervention effects on patient-reported outcomes. [file 13058_2022_1530_MOESM1_ESM.docx]

**Additional file 1**

- **Table S1.** Intervention effects on cognitive functioning.
- **Table S2.** Intervention effects on cognitive functioning, per protocol.
- **Table S3.** Intervention effects, separately for patients with and without endocrine therapy.
- **Table S4.** Intervention effects, separately for patients of different age categories (30-44, 45-59, 60-75 years).
- **Table S5.** Intervention effects, separately for patients of with pre- and peri- menopausal status and patients with postmenopausal status.
- **Table S6.** Intervention effects on cognitive functioning, stratified for low versus high levels of fatigue measured with the EORTC QLQ C-30 fatigue scale.
- **Table S7.** Intervention effects on patient-reported outcomes.

**Table S1.** Intervention effects on cognitive functioning.

| **Cognitive domain** | **Outcome measures** | **Intervention** | **Control** | **Treatment effect† (95% CI)** | **Effect Size‡** |
| --- | --- | --- | --- | --- | --- |
| **Learning and memory** | HVLT-R total recall |  |  |  |  |
|  | *n*  Baseline  Follow-up | *82*  26.6 (4.4)  25.9 (4.3) | *82*  26.7 (4.3)  25.8 (4.7) | 0.14 (-1.01; 1.29) | 0.03 |
|  | HVLT-R delayed recall |  |  |  |  |
|  | *n*  Baseline  Follow-up | *82*  8.9 (2.2)  8.8 (2.4) | *81*  9.3 (2.2)  9.3 (2.3) | -0.17 (-0.74; 0.40) | -0.08 |
|  | HVLT-R recognition |  |  |  |  |
|  | *n*  Baseline  Follow-up | *82*  10.7 (1.5)  10.7 (1.4) | *82*  10.6 (1.4)  10.6 (1.6) | 0.01 (-0.42; 0.44) | 0.01 |
|  | Wordlist Learning |  |  |  |  |
|  | *n*  Baseline  Follow-up | *84*  41.8 (9.6)  48.9 (8.8) | *83*  43.0 (9.8)  48.5 (10.8) | 1.33 (-0.80; 3.46) | 0.14 |
|  | Wordlist Delayed Recall |  |  |  |  |
|  | *n*  Baseline  Follow-up | *76*  8.5 (2.6)  10.5 (2.7) | *80*  9.1 (2.7)  10.9 (2.9) | -0.01 (-0.73; 0.70) | 0.00 |
|  | Wordlist Recognition |  |  |  |  |
|  | *n*  Baseline  Follow-up | *79*  43.0 (2.6)  43.8 (1.6) | *82*  42.8 (2.9)  43.3 (2.7) | 0.49 (-0.12; 1.09) | 0.18 |
| **Attention and working memory** | Box Tapping |  |  |  |  |
|  | *n*  Baseline  Follow-up | *78*  8.6 (1.9)  8.7 (2.0) | *78*  8.6 (2.1)  9.3 (2.0) | -0.63 (-1.20; -0.07) | -0.31 |
|  | Digit Sequences I |  |  |  |  |
|  | *n*  Baseline  Follow-up | *84*  8.8 (2.0)  9.5 (2.4) | *84*  7.8 (1.9)  8.6 (2.2) | 0.34 (-0.28; 0.96) | 0.17 |
|  | Digit Sequences II |  |  |  |  |
|  | *n*  Baseline  Follow-up | *84*  6.5 (2.6)  7.6 (3.0) | *84*  6.0 (1.9)  7.0 (2.4) | 0.39 (-0.29; 1.08) | 0.17 |
| **Processing speed** | Reaction Time |  |  |  |  |
|  | *n*  Baseline  Follow-up | *73*  378 (60)  333 (61) | *78*  372 (64)  340 (65) | -11.3 (-28.5; 6.0) | 0.18 |
|  | Connecting the Dots I |  |  |  |  |
|  | *n*  Baseline  Follow-up | *79*  40.2 (9.9)  36.0 (8.1) | *79*  38.6 (9.3)  35.5 (8.9) | -0.27 (-2.34; 1.80) | 0.03 |
| **Executive functioning** | Connecting the Dots II |  |  |  |  |
|  | *n*  Baseline  Follow-up | *77*  67.8 (17.3)  60.9 (18.3) | *81*  68.8 (22.8)  63.0 (18.4) | -1.53 (-5.71; 2.66) | 0.08 |
|  | Place the Beads |  |  |  |  |
|  | *n*  Baseline  Follow-up | *82*  31.4 (17.2)  26.4 (14.6) | *83*  30.7 (18.8)  26.3 (15.7) | -0.16 (-4.22; 3.90) | 0.01 |
| **Motor functioning** | Fill the Grid |  |  |  |  |
|  | *n*  Baseline  Follow-up | *82*  73.8 (15.5)  68.2 (12.1) | *83*  72.5 (13.7)  68.6 (13.5) | -1.28 (-4.07; 1.51) | 0.09 |
| **Self-reported cognitive functioning** | MDASI-MM Severity |  |  |  |  |
|  | *n*  Baseline  Follow-up | *84*  6.0 (1.9)  4.8 (2.3) | *86*  5.6 (1.8)  5.3 (1.8) | -0.68 (-1.23; -0.12) | 0.36 |
|  | MDASI-MM Interference |  |  |  |  |
|  | *n*  Baseline  Follow-up | *84*  3.6 (2.0)  2.7 (2.1) | *86*  3.4 (1.9)  3.0 (2.1) | -0.42 (-0.93; 0.09) | 0.22 |

Abbreviations: HVLT-R, Hopkins Verbal Learning Test-Revised; MDASI-MM, MD Anderson Symptom Inventory for multiple myeloma.

Values indicate mean (SD).

† The intervention effect is the regression coefficient of a linear regression analysis adjusted for baseline, age and endocrine therapy.

‡ Effect Sizes (ES) were calculated by dividing Beta by the pooled SD at baseline, with positive ESs meaning a beneficial effect of the intervention on a specific outcome. ESs <0.2 indicate “no difference”, ESs between 0.2 and 0.5 indicate “small differences”, ESs between 0.5 and 0.8 indicate “medium differences” and ESs ≥ 0.8 indicate “large differences” [1]. An ES of 0.5 or higher was considered clinically relevant [2].

Note: tests/questionnaires for which a higher score indicates worse performance/functioning/more symptoms: Reaction Time, Connecting the Dots (I & II), Place the Beads, Fill the Grid and MDASI-MM (Severity and Interference).

**Table S2.** Intervention effects on cognitive functioning, per protocol.

| **Cognitive domain** | **Outcome measures** | **Intervention** | **Control** | **Treatment effect† (95% CI)** | **Effect Size‡** |
| --- | --- | --- | --- | --- | --- |
| **Learning and memory** | HVLT-R total recall |  |  |  |  |
|  | *n*  Baseline  Follow-up | *61*  26.1 (4.3)  26.4 (4.2) | *82*  26.7 (4.3)  25.8 (4.7) | 0.99 (-0.23; 2.21) | 0.23 |
|  | HVLT-R delayed recall |  |  |  |  |
|  | *n*  Baseline  Follow-up | *61*  8.6 (2.1)  8.9 (2.4) | *81*  9.3 (2.2)  9.3 (2.3) | 0.20 (-0.40; 0.80) | 0.09 |
|  | HVLT-R recognition |  |  |  |  |
|  | *n*  Baseline  Follow-up | *61*  10.6 (1.6)  10.8 (1.3) | *82*  10.6 (1.4)  10.6 (1.6) | 0.15 (-0.31; 0.62) | 0.11 |
|  | Wordlist Learning |  |  |  |  |
|  | *n*  Baseline  Follow-up | *61*  41.8 (9.1)  49.5 (7.3) | *83*  43.0 (9.8)  48.5 (10.8) | 1.85 (-0.49; 4.19) | 0.19 |
|  | Wordlist Delayed Recall |  |  |  |  |
|  | *n*  Baseline  Follow-up | *55*  8.5 (2.4)  10.7 (2.3) | *80*  9.1 (2.7)  10.9 (2.9) | 0.12 (-0.66; 0.89) | 0.05 |
|  | Wordlist Recognition |  |  |  |  |
|  | *n*  Baseline  Follow-up | *57*  43.1 (2.3)  43.9 (1.4) | *82*  42.8 (2.9)  43.3 (2.7) | 0.59 (-0.10; 1.27) | 0.22 |
| **Attention and working memory** | Box Tapping |  |  |  |  |
|  | *n*  Baseline  Follow-up | *57*  8.4 (2.0)  8.4 (2.1) | *78*  8.6 (2.1)  9.3 (2.0) | -0.76 (-1.40; -0.12) | -0.36 |
|  | Digit Sequences I |  |  |  |  |
|  | *n*  Baseline  Follow-up | *61*  8.6 (2.0)  9.4 (2.6) | *84*  7.8 (1.9)  8.6 (2.2) | 0.32 (-0.38; 1.03) | 0.17 |
|  | Digit Sequences II |  |  |  |  |
|  | *n*  Baseline  Follow-up | *61*  6.5 (2.6)  7.6 (3.0) | *84*  6.0 (1.9)  7.0 (2.4) | 0.39 (-0.34; 1.11) | 0.17 |
| **Processing speed** | Reaction Time |  |  |  |  |
|  | *n*  Baseline  Follow-up | *53*  380 (58)  330 (57) | *78*  372 (64)  340 (65) | -16.19 (-35.39; 3.00) | 0.26 |
|  | Connecting the Dots I |  |  |  |  |
|  | *n*  Baseline  Follow-up | *57*  40.9 (9.8)  36.5 (8.5) | *79*  38.6 (9.3)  35.5 (8.9) | -0.32 (-2.65; 2.01) | 0.03 |
| **Executive functioning** | Connecting the Dots II |  |  |  |  |
|  | *n*  Baseline  Follow-up | *56*  68.1 (17.3)  61.4 (17.7) | *81*  68.8 (22.8)  63.0 (18.4) | -1.72 (-6.30; 2.87) | 0.08 |
|  | Place the Beads |  |  |  |  |
|  | *n*  Baseline  Follow-up | *59*  32.7 (16.7)  27.8 (15.4) | *83*  30.7 (18.8)  26.3 (15.7) | 0.58 (-3.96; 5.13) | -0.03 |
| **Motor functioning** | Fill the Grid |  |  |  |  |
|  | *n*  Baseline  Follow-up | *59*  73.5 (16.4)  67.7 (12.6) | *83*  72.5 (13.7)  68.6 (13.5) | -2.08 (-5.13; 0.97) | 0.14 |
| **Self-reported cognitive functioning** | MDASI-MM Severity |  |  |  |  |
|  | *n*  Baseline  Follow-up | *61*  5.9 (2.1)  4.7 (2.2) | *86*  5.6 (1.8)  5.3 (1.8) | -0.62 (-1.20; -0.05) | 0.32 |
|  | MDASI-MM Interference |  |  |  |  |
|  | *n*  Baseline  Follow-up | *61*  3.5 (2.1)  2.7 (2.2) | *86*  3.4 (1.9)  3.0 (2.1) | -0.36 (-0.94; 0.22) | 0.19 |

Abbreviations: HVLT-R, Hopkins Verbal Learning Test-Revised; MDASI-MM, MD Anderson Symptom Inventory for multiple myeloma.

Values indicate mean (SD).

† The intervention effect is the regression coefficient of a linear regression analysis adjusted for baseline, age and endocrine therapy.

‡ Effect Sizes (ES) were calculated by dividing Beta by the pooled SD at baseline, with positive ESs meaning a beneficial effect of the intervention on a specific outcome. ESs <0.2 indicate “no difference”, ESs between 0.2 and 0.5 indicate “small differences”, ESs between 0.5 and 0.8 indicate “medium differences” and ESs ≥ 0.8 indicate “large differences” [1]. An ES of 0.5 or higher was considered clinically relevant [2].

Note: tests/questionnaires for which a higher score indicates worse performance/functioning/more symptoms: Reaction Time, Connecting the Dots (I & II), Place the Beads, Fill the Grid and MDASI-MM (Severity and Interference).

**Table S3.** Intervention effects, separately for patients with and without endocrine therapy.

| **Cognitive domain** | **Outcome measures** | | **Intervention** | **Control** | **Treatment effect† (95% CI)** | **Effect Size‡** |
| --- | --- | --- | --- | --- | --- | --- |
| **Learning and memory** | HVLT-R total recall | |  |  |  |  |
|  | ET-yes | *n*  Baseline  Follow-up | *53*  26.3 (4.6)  25.4 (4.8) | *50*  25.9 (4.4)  25.2 (5.0) | -0.05 (-1.59; 1.49) | -0.01 |
|  | ET-no | *n*  Baseline  Follow-up | *29*  27.1 (3.8)  26.7 (3.2) | *32*  28.0 (3.9)  26.8 (4.2) | 0.28 (-1.46; 2.03) | 0.07 |
|  | HVLT-R delayed recall | |  |  |  |  |
|  | ET-yes | *n*  Baseline  Follow-up | *53*  8.9 (2.2)  8.6 (2.6) | *49*  9.1 (2.2)  8.9 (2.5) | -0.06 (-0.83; 0.70) | -0.03 |
|  | ET-no | *n*  Baseline  Follow-up | 29  9.0 (2.1)  9.1 (2.0) | 32  9.7 (2.1)  9.9 (2.0) | -0.44 (-1.29; 0.41) | -0.21 |
|  | HVLT-R recognition | |  |  |  |  |
|  | ET-yes | *n*  Baseline  Follow-up | *53*  10.6 (1.5)  10.6 (1.5) | *50*  10.5 (1.4)  10.4 (1.8) | 0.18 (-0.39; 0.76) | 0.13 |
|  | ET-no | *n*  Baseline  Follow-up | *29*  10.9 (1.5)  10.8 (1.2) | *32*  10.8 (1.4)  11.0 (1.3) | -0.30 (-0.92; 0.32) | -0.21 |
|  | Wordlist Learning | |  |  |  |  |
|  | ET-yes | *n*  Baseline  Follow-up | *53*  41.0 (10.6)  48.5 (9.4) | *49*  41.6 (10.5)  47.0 (11.3) | 1.79 (-1.06; 4.64) | 0.17 |
|  | ET-no | *n*  Baseline  Follow-up | *31*  43.2 (7.5)  49.7 (7.9) | *34*  45.1 (8.4)  50.6 (9.8) | 0.65 (-2.58; 3.89) | 0.08 |
|  | Wordlist Delayed Recall | |  |  |  |  |
|  | ET-yes | *n*  Baseline  Follow-up | *48*  8.2 (2.6)  10.3 (2.7) | *46*  9.0 (2.9)  10.9 (2.9) | -0.12 (-1.06; 0.82) | -0.04 |
|  | ET-no | *n*  Baseline  Follow-up | *28*  9.0 (2.4)  10.9 (2.5) | *34*  9.2 (2.3)  10.9 (3.0) | 0.12 (-1.02; 1.27) | 0.05 |
|  | Wordlist Recognition | |  |  |  |  |
|  | ET-yes | *n*  Baseline  Follow-up | *50*  42.7 (2.7)  43.6 (1.8) | *48*  42.9 (2.8)  43.2 (2.7) | 0.50 (-0.30; 1.30) | 0.18 |
|  | ET-no | *n*  Baseline  Follow-up | *29*  43.4 (2.4)  44.1 (1.2) | *34*  42.8 (3.0)  43.4 (2.7) | 0.50 (-0.47; 1.47) | 0.18 |
| **Attention and working memory** | Box Tapping | |  |  |  |  |
|  | ET-yes | *n*  Baseline  Follow-up | *49*  8.5 (2.1)  8.6 (2.2) | *48*  8.5 (2.0)  9.1 (2.2) | -0.47 (-1.25; 0.31) | -0.23 |
|  | ET-no | *n*  Baseline  Follow-up | *59*  8.8 (1.5)  8.8 (1.5) | *30*  8.7 (2.4)  9.7 (1.6) | -0.92 (-1.69; -0.16) | -0.46 |
|  | Digit Sequences I | |  |  |  |  |
|  | ET-yes | *n*  Baseline  Follow-up | *53*  8.9 (2.2)  9.8 (2.7) | *49*  8.1 (2.1)  8.4 (2.1) | 0.85 (0.08; 1.63) | 0.39 |
|  | ET-no | *n*  Baseline  Follow-up | *31*  8.5 (1.7)  9.1 (1.9) | *35*  7.5 (1.4)  8.9 (2.3) | -0.49 (-1.53; 0.56) | -0.30 |
|  | Digit Sequences II | |  |  |  |  |
|  | ET-yes | *n*  Baseline  Follow-up | *53*  6.7 (2.8)  7.8 (3.0) | *49*  5.8 (1.8)  6.7 (2.5) | 0.49 (-0.38; 1.36) | 0.20 |
|  | ET-no | *n*  Baseline  Follow-up | *31*  6.2 (2.4)  7.3 (3.0) | *35*  6.4 (2.0)  7.3 (2.2) | 0.13 (-1.03; 1.29) | 0.06 |
| **Processing speed** | Reaction Time | |  |  |  |  |
|  | ET-yes | *n*  Baseline  Follow-up | *44*  377 (62)  333 (54) | *43*  368 (65)  343 (65) | -16.0 (-36.0; 3.9) | 0.25 |
|  | ET-no | *n*  Baseline  Follow-up | *29*  379 (57)  333 (72) | *35*  376 (65)  335 (66) | -3.9 (-34.1; 26.4) | 0.06 |
|  | Connecting the Dots I | |  |  |  |  |
|  | ET-yes | *n*  Baseline  Follow-up | *49*  41.8 (10.5)  36.7 (9.3) | *44*  40.2 (8.1)  35.6 (8.0) | 0.43 (-2.42; 3.28) | -0.05 |
|  | ET-no | *n*  Baseline  Follow-up | *30*  37.6 (8.3)  34.9 (5.6) | *35*  36.6 (10.5)  35.4 (10.0) | -1.18 (-4.20; 1.84) | 0.12 |
| **Executive functioning** | Connecting the Dots II | |  |  |  |  |
|  | ET-yes | *n*  Baseline  Follow-up | *48*  67.4 (16.1)  62.0 (20.4) | *47*  71.6 (21.0)  65.4 (19.0) | -0.95 (-6.75; 4.86) | 0.05 |
|  | ET-no | *n*  Baseline  Follow-up | *29*  68.4 (19.4)  59.0 (14.4) | *34*  64.9 (24.9)  59.6 (17.4) | -1.43 (-7.40; 4.54) | 0.06 |
|  | Place the Beads | |  |  |  |  |
|  | ET-yes | *n*  Baseline  Follow-up | *51*  30.8 (16.0)  26.7 (14.9) | *49*  33.6 (20.4)  28.3 (17.6) | -0.47 (-6.20; 5.26) | 0.03 |
|  | ET-no | *n*  Baseline  Follow-up | *31*  32.4 (19.2)  26.0 (14.3) | *34*  26.6 (15.6)  23.3 (12.1) | 0.57 (-5.18; 6.32) | -0.03 |
| **Motor functioning** | Fill the Grid | |  |  |  |  |
|  | ET-yes | *n*  Baseline  Follow-up | *52*  74.3 (14.7)  69.7 (11.7) | *48*  72.7 (11.9)  69.0 (14.0) | -0.21 (-3.77; 3.35) | 0.02 |
|  | ET-no | *n*  Baseline  Follow-up | *30*  72.9 (17.1)  65.7 (12.5) | *35*  72.2 (16.0)  68.2 (13.0) | -2.87 (-7.44; 1.70) | 0.17 |
| **Self-reported cognitive functioning** | MDASI-MM Severity | |  |  |  |  |
|  | ET-yes | *n*  Baseline  Follow-up | *53*  6.2 (2.0)  4.8 (2.3) | *52*  5.6 (1.9)  5.2 (1.8) | -0.72 (-1.45; 0.004) | 0.37 |
|  | ET-no | *n*  Baseline  Follow-up | *31*  5.6 (1.8)  4.7 (2.3) | *34*  5.6 (1.7)  5.3 (1.8) | -0.58 (-1.48; 0.32) | 0.33 |
|  | MDASI-MM Interference | |  |  |  |  |
|  | ET-yes | *n*  Baseline  Follow-up | *53*  3.7 (2.0)  2.5 (2.0) | *52*  3.1 (1.9)  2.8 (2.1) | -0.68 (-1.34; -0.01) | 0.35 |
|  | ET-no | *n*  Baseline  Follow-up | *31*  3.3 (2.0)  3.0 (2.3) | *34*  3.7 (1.8)  3.2 (2.2) | 0.09 (-0.74; 0.92) | -0.05 |

Abbreviations: ET, endocrine therapy; HVLT-R, Hopkins Verbal Learning Test-Revised; MDASI-MM, MD Anderson Symptom Inventory for multiple myeloma.

Values indicate mean (SD).

† The intervention effect is the regression coefficient of a linear regression analysis adjusted for baseline and age.

‡ Effect Sizes (ES) were calculated by dividing Beta by the pooled SD at baseline, with positive ESs meaning a beneficial effect of the intervention on a specific outcome. ESs <0.2 indicate “no difference”, ESs between 0.2 and 0.5 indicate “small differences”, ESs between 0.5 and 0.8 indicate “medium differences” and ESs ≥ 0.8 indicate “large differences” [1]. An ES of 0.5 or higher was considered clinically relevant [2].

Note: tests/questionnaires for which a higher score indicates worse performance/functioning/more symptoms: Reaction Time, Connecting the Dots (I & II), Place the Beads, Fill the Grid and MDASI-MM (Severity and Interference).

**Table S4.** Intervention effects, separately for patients of different age categories (30-44, 45-59, 60-75 years).

| **Cognitive domain** | **Outcome measures** | | **Intervention** | **Control** | **Treatment effect† (95% CI)** | **Effect Size‡** |
| --- | --- | --- | --- | --- | --- | --- |
| **Learning and memory** | HVLT-R total recall | |  |  |  |  |
|  | Age (30-44 yrs) | *n*  Baseline  Follow-up | *15*  26.7 (4.3)  27.1 (4.1) | *16*  27.2 (4.2)  26.8 (3.3) | 0.57 (-1.81; 2.95) | 0.14 |
|  | Age (45-59 yrs) | *n*  Baseline  Follow-up | *49*  27.4 (4.2)  26.0 (4.3) | *48*  27.0 (4.4)  26.3 (5.1) | -0.55 (-2.16; 1.06) | -0.13 |
|  | Age (60-75 yrs) | *n*  Baseline  Follow-up | *18*  24.2 (4.2)  24.6 (4.6) | *18*  25.5 (4.1)  23.8 (4.5) | 1.71 (-0.52; 3.95) | 0.41 |
|  | HVLT-R delayed recall | |  |  |  |  |
|  | Age (30-44 yrs) | *n*  Baseline  Follow-up | *15*  9.1 (2.5)  9.0 (2.9) | *16*  9.3 (2.5)  10.1 (1.6) | -0.91 (-2.12; 0.30) | -0.37 |
|  | Age (45-59 yrs) | *n*  Baseline  Follow-up | *49*  9.3 (1.9)  9.1 (2.2) | *48*  9.5 (2.0)  9.2 (2.6) | 0.10 (-0.66; 0.87) | 0.05 |
|  | Age (60-75 yrs) | *n*  Baseline  Follow-up | *18*  7.7 (2.2)  7.8 (2.3) | *17*  8.8 (2.2)  8.8 (2.0) | -0.53 (-1.76; 0.70) | -0.23 |
|  | HVLT-R recognition | |  |  |  |  |
|  | Age (30-44 yrs) | *n*  Baseline  Follow-up | *15*  10.5 (1.5)  10.8 (1.7) | *16*  10.3 (1.7)  10.8 (1.0) | 0.00 (-1.04; 1.04) | 0.00 |
|  | Age (45-59 yrs) | *n*  Baseline  Follow-up | *49*  10.9 (1.4)  10.7 (1.3) | *48*  10.9 (1.2)  10.6 (1.7) | 0.05 (-0.50; 0.59) | 0.04 |
|  | Age (60-75 yrs) | *n*  Baseline  Follow-up | *18*  10.1 (1.8)  10.6 (1.3) | *18*  10.4 (1.4)  10.7 (2.0) | -0.07 (-1.06; 0.92) | -0.05 |
|  | Wordlist Learning | |  |  |  |  |
|  | Age (30-44 yrs) | *n*  Baseline  Follow-up | *15*  40.3 (10.5)  49.9 (8.6) | *17*  44.1 (7.9)  51.2 (9.4) | 0.88 (-3.67; 5.43) | 0.10 |
|  | Age (45-59 yrs) | *n*  Baseline  Follow-up | *49*  42.2 (10.2)  49.5 (8.4) | *48*  43.9 (9.9)  48.9 (10.5) | 1.62 (-1.14; 4.39) | 0.16 |
|  | Age (60-75 yrs) | *n*  Baseline  Follow-up | *20*  42.0 (7.4)  46.9 (10.1) | *18*  39.7 (10.9)  44.7 (12.2) | -0.05 (-4.62; 4.52) | -0.01 |
|  | Wordlist Delayed Recall | |  |  |  |  |
|  | Age (30-44 yrs) | *n*  Baseline  Follow-up | *13*  8.7 (2.6)  10.9 (2.3) | *17*  8.9 (2.3)  11.1 (2.9) | -0.12 (-1.73; 1.48) | -0.05 |
|  | Age (45-59 yrs) | *n*  Baseline  Follow-up | *46*  8.2 (2.8)  10.5 (2.8) | *46*  9.3 (2.6)  11.2 (2.8) | -0.04 (-0.98; 0.90) | -0.02 |
|  | Age (60-75 yrs) | *n*  Baseline  Follow-up | *17*  9.1 (1.9)  10.2 (2.8) | *17*  8.7 (3.1)  9.9 (3.3) | -0.10 (-1.78; 1.58) | -0.04 |
|  | Wordlist Recognition | |  |  |  |  |
|  | Age (30-44 yrs) | *n*  Baseline  Follow-up | *14*  43.4 (3.0)  44.1 (1.7) | *17*  43.0 (3.4)  44.1 (1.3) | 0.01 (-1.11; 1.12) | 0.00 |
|  | Age (45-59 yrs) | *n*  Baseline  Follow-up | *47*  42.9 (2.8)  43.8 (1.7) | *48*  43.0 (2.5)  43.6 (2.2) | 0.29 (-0.41; 0.99) | 0.11 |
|  | Age (60-75 yrs) | *n*  Baseline  Follow-up | *18*  42.7 (1.7)  43.4 (1.3) | *17*  42.1 (3.3)  41.7 (4.0) | 1.32 (-0.28; 2.92) | 0.51 |
| **Attention and working memory** | Box Tapping | |  |  |  |  |
|  | Age (30-44 yrs) | *n*  Baseline  Follow-up | *15*  9.0 (1.9)  9.3 (1.8) | *16*  9.6 (1.7)  10.6 (1.6) | -1.09 (-2.30; 0.12) | -0.61 |
|  | Age (45-59 yrs) | *n*  Baseline  Follow-up | *46*  8.5 (2.1)  8.7 (1.6) | *47*  8.3 (2.2)  9.1 (1.9) | -0.38 (-1.05; 0.29) | -0.18 |
|  | Age (60-75 yrs) | *n*  Baseline  Follow-up | *17*  8.6 (1.6)  7.9 (2.8) | *15*  8.3 (2.4)  8.7 (2.1) | -0.79 (-2.63; 1.04) | -0.40 |
|  | Digit Sequences I | |  |  |  |  |
|  | Age (30-44 yrs) | *n*  Baseline  Follow-up | *15*  8.8 (1.8)  9.8 (2.4) | *17*  8.5 (1.5)  9.3 (1.8) | 0.28 (-1.12; 1.68) | 0.17 |
|  | Age (45-59 yrs) | *n*  Baseline  Follow-up | *49*  8.9 (2.4)  9.5 (2.5) | *49*  7.8 (1.8)  8.6 (2.4) | 0.15 (-0.68; 0.98) | 0.07 |
|  | Age (60-75 yrs) | *n*  Baseline  Follow-up | *20*  8.4 (1.3)  9.3 (2.4) | *18*  7.4 (2.2)  8.1 (1.7) | 0.87 (-0.55; 2.29) | 0.48 |
|  | Digit Sequences II | |  |  |  |  |
|  | Age (30-44 yrs) | *n*  Baseline  Follow-up | *15*  6.7 (2.4)  8.6 (3.1) | *17*  6.9 (2.1)  8.3 (2.3) | 0.39 (-1.54; 2.31) | 0.18 |
|  | Age (45-59 yrs) | *n*  Baseline  Follow-up | *49*  6.3 (2.8)  7.3 (3.0) | *49*  6.0 (1.9)  6.6 (2.3) | 0.63 (-0.26; 1.52) | 0.27 |
|  | Age (60-75 yrs) | *n*  Baseline  Follow-up | *20*  6.9 (2.6)  7.6 (2.9) | *18*  5.3 (1.4)  6.8 (2.3) | -0.58 (-1.91; 0.76) | -0.25 |
| **Processing speed** | Reaction Time | |  |  |  |  |
|  | Age (30-44 yrs) | *n*  Baseline  Follow-up | *12*  364 (49)  300 (37.5) | *16*  349 (53)  322 (40) | -27.8 (-54.9; -0.6) | 0.55 |
|  | Age (45-59 yrs) | *n*  Baseline  Follow-up | *41*  381 (61)  329 (52) | *45*  368 (59)  327 (47) | -4.4 (-22.8; 14.0) | 0.07 |
|  | Age (60-75 yrs) | *n*  Baseline  Follow-up | *20*  382 (65)  361 (79) | *17*  404 (80)  389 (97) | -17.1 (-71.7; 37.5) | 0.24 |
|  | Connecting the Dots I | |  |  |  |  |
|  | Age (30-44 yrs) | *n*  Baseline  Follow-up | *13*  32.8 (5.2)  31.2 (5.1) | *16*  38.0 (9.3)  30.2 (5.9) | 3.50 (-0.01; 7.00) | -0.44 |
|  | Age (45-59 yrs) | *n*  Baseline  Follow-up | *46*  39.2 (9.2)  34.3 (6.9) | *47*  36.9 (8.2)  34.1 (7.2) | -0.64 (-3.27; 1.99) | 0.07 |
|  | Age (60-75 yrs) | *n*  Baseline  Follow-up | *20*  47.4 (9.5)  43.3 (7.9) | *16*  44.0 (10.9)  44.8 (9.3) | -2.68 (-8.27; 2.91) | 0.26 |
| **Executive functioning** | Connecting the Dots II | |  |  |  |  |
|  | Age (30-44 yrs) | *n*  Baseline  Follow-up | *13*  57.3 (9.4)  48.4 (8.4) | *14*  59.3 (18.9)  48.9 (8.2) | 0.31 (-5.73; 6.34) | -0.02 |
|  | Age (45-59 yrs) | *n*  Baseline  Follow-up | *47*  66.0 (18.2)  58.7 (16.2) | *49*  64.1 (16.7)  60.1 (15.6) | -2.48 (-7.41; 2.46) | 0.14 |
|  | Age (60-75 yrs) | *n*  Baseline  Follow-up | *17*  80.8 (11.2)  76.4 (19.6) | *18*  88.8 (28.7)  81.9 (17.3) | -2.64 (-15.22; 9.93) | 0.12 |
|  | Place the Beads | |  |  |  |  |
|  | Age (30-44 yrs) | *n*  Baseline  Follow-up | *15*  25.1 (15.6)  21.7 (11.1) | *16*  24.6 (13.1)  20.6 (12.7) | 0.54 (-7.24; 8.31) | -0.04 |
|  | Age (45-59 yrs) | *n*  Baseline  Follow-up | *48*  28.6 (14.1)  26.5 (15.0) | *49*  28.3 (17.1)  26.5 (15.1) | -0.11 (-5.72; 5.50) | 0.01 |
|  | Age (60-75 yrs) | *n*  Baseline  Follow-up | *19*  43.4 (20.5)  30.0 (15.5) | *18*  42.8 (22.7)  30.7 (18.6) | -1.00 (-10.36; 8.36) | 0.05 |
| **Motor functioning** | Fill the Grid | |  |  |  |  |
|  | Age (30-44 yrs) | *n*  Baseline  Follow-up | *15*  66.2 (12.6)  60.8 (10.1) | *17*  68.6 (11.8)  59.9 (9.9) | 1.56 (-5.79; 8.91) | -0.13 |
|  | Age (45-59 yrs) | *n*  Baseline  Follow-up | *47*  73.3 (15.2)  67.9 (11.3) | *49*  72.1 (14.0)  68.5 (12.1) | -1.38 (-4.76; 2.01) | 0.09 |
|  | Age (60-75 yrs) | *n*  Baseline  Follow-up | *20*  80.5 (16.0)  74.7 (12.2) | *17*  77.7 (13.9)  77.8 (15.2) | -5.12 (-11.18; 0.93) | 0.34 |
| **Self-reported cognitive functioning** | MDASI-MM Severity | |  |  |  |  |
|  | Age (30-44 yrs) | *n*  Baseline  Follow-up | *15*  5.2 (1.7)  4.6 (2.3) | *17*  6.0 (1.8)  6.1 (1.7) | -1.38 (-2.86; 0.09) | 0.78 |
|  | Age (45-59 yrs) | *n*  Baseline  Follow-up | *49*  6.2 (1.9)  4.9 (2.3) | *51*  5.6 (1.6)  5.2 (1.7) | -0.66 (-1.39; 0.07) | 0.37 |
|  | Age (60-75 yrs) | *n*  Baseline  Follow-up | *20*  6.1 (2.1)  4.7 (2.2) | *18*  5.3 (2.3)  4.6 (1.9) | -0.37 (-1.62; 0.87) | 0.17 |
|  | MDASI-MM Interference | |  |  |  |  |
|  | Age (30-44 yrs) | *n*  Baseline  Follow-up | *15*  3.7 (1.7)  3.0 (2.3) | *17*  3.9 (1.7)  4.3 (2.0) | -1.12 (-2.47; 0.23) | 0.66 |
|  | Age (45-59 yrs) | *n*  Baseline  Follow-up | *49*  3.7 (1.9)  2.8 (2.1) | *51*  3.1 (1.7)  2.6 (1.9) | -0.21 (-0.85; 0.42) | 0.12 |
|  | Age (60-75 yrs) | *n*  Baseline  Follow-up | *20*  3.2 (2.4)  2.1 (2.1) | *18*  3.6 (2.4)  2.8 (2.5) | -0.41 (-1.67; 0.86) | 0.17 |

Abbreviations: HVLT-R, Hopkins Verbal Learning Test-Revised; MDASI-MM, MD Anderson Symptom Inventory for multiple myeloma.

Values indicate mean (SD).

† The intervention effect is the regression coefficient of a linear regression analysis adjusted for baseline and endocrine therapy.

‡ Effect Sizes (ES) were calculated by dividing Beta by the pooled SD at baseline, with positive ESs meaning a beneficial effect of the intervention on a specific outcome. ESs <0.2 indicate “no difference”, ESs between 0.2 and 0.5 indicate “small differences”, ESs between 0.5 and 0.8 indicate “medium differences” and ESs ≥ 0.8 indicate “large differences” [1]. An ES of 0.5 or higher was considered clinically relevant [2].

Note: tests/questionnaires for which a higher score indicates worse performance/functioning/more symptoms: Reaction Time, Connecting the Dots (I & II), Place the Beads, Fill the Grid and MDASI-MM (Severity and Interference).

**Table S5.** Intervention effects, separately for patients of with pre- and peri- menopausal status and patients with postmenopausal status.

| **Cognitive domain** | **Outcome measures** | | **Intervention** | **Control** | **Treatment effect† (95% CI)** | **Effect Size‡** |
| --- | --- | --- | --- | --- | --- | --- |
| **Learning and memory** | HVLT-R total recall | |  |  |  |  |
|  | Pre- and peri- | *n*  Baseline  Follow-up | *9*  28.1 (3.3)  28.7 (3.2) | *10*  28.1 (4.1)  28.3 (3.2) | 0.22 (-2.99; 3.43) | 0.06 |
|  | Post | *n*  Baseline  Follow-up | *73*  26.4 (4.4)  25.5 (4.3) | *72*  26.5 (4.3)  25.5 (4.8) | 0.13 (-1.12; 1.37) | 0.03 |
|  | HVLT-R delayed recall | |  |  |  |  |
|  | Pre- and peri- | *n*  Baseline  Follow-up | *9*  10.0 (1.3)  10.0 (2.3) | *10*  10.1 (1.8)  10.6 (1.4) | -0.44 (-2.11; 1.22) | -0.29 |
|  | Post | *n*  Baseline  Follow-up | *73*  8.8 (2.2)  8.7 (2.4) | *71*  9.2 (2.2)  9.1 (2.4) | -0.13 (-0.75; 0.49) | -0.06 |
|  | HVLT-R recognition | |  |  |  |  |
|  | Pre- and peri- | *n*  Baseline  Follow-up | *9*  10.9 (1.5)  11.3 (1.0) | *10*  10.4 (1.8)  11.3 (0.7) | 0.21 (-0.66; 1.09) | 0.13 |
|  | Post | *n*  Baseline  Follow-up | *73*  10.7 (1.5)  10.6 (1.4) | *72*  10.7 (1.3)  10.6 (1.7) | 0.05 (-0.41; 0.51) | 0.04 |
|  | Wordlist Learning | |  |  |  |  |
|  | Pre- and peri- | *n*  Baseline  Follow-up | *9*  40.1 (9.9)  50.9 (8.5) | *11*  44.6 (6.8)  51.1 (7.7) | 1.82 (-5.21; 8.84) | 0.21 |
|  | Post | *n*  Baseline  Follow-up | *75*  42.0 (9.6)  48.7 (8.9) | *72*  42.8 (10.2)  48.1 (11.1) | 1.16 (-1.12; 3.43) | 0.12 |
|  | Wordlist Delayed Recall | |  |  |  |  |
|  | Pre- and peri- | *n*  Baseline  Follow-up | *8*  8.4 (2.9)  10.9 (2.6) | *11*  8.6 (1.7)  11.2 (2.8) | -0.23 (-2.24; 1.78) | -0.10 |
|  | Post | *n*  Baseline  Follow-up | *68*  8.5 (2.6)  10.5 (2.7) | *69*  9.1 (2.8)  10.8 (3.0) | 0.01 (-0.77; 0.79) | 0.00 |
|  | Wordlist Recognition | |  |  |  |  |
|  | Pre- and peri- | *n*  Baseline  Follow-up | *9*  43.1 (3.5)  44.3 (2.0) | *11*  43.1 (3.5)  44.3 (1.5) | 0.18 (-1.70; 2.06) | 0.05 |
|  | Post | *n*  Baseline  Follow-up | *70*  42.9 (2.5)  43.7 (1.5) | *71*  42.8 (2.8)  43.1 (2.8) | 0.53 (-0.11; 1.17) | 0.20 |
| **Attention and working memory** | Box Tapping | |  |  |  |  |
|  | Pre- and peri- | *n*  Baseline  Follow-up | *9*  9.2 (1.6)  9.6 (1.6) | *10*  9.3 (1.2)  10.9 (1.6) | -1.40 (-3.07; 0.26) | -1.06 |
|  | Post | *n*  Baseline  Follow-up | *69*  8.5 (2.0)  8.6 (2.0) | *68*  8.5 (2.2)  9.1 (1.9) | -0.55 (-1.16; 0.07) | -0.26 |
|  | Digit Sequences I | |  |  |  |  |
|  | Pre- and peri- | *n*  Baseline  Follow-up | *9*  9.2 (2.3)  9.6 (2.8) | *11*  8.2 (1.2)  9.5 (2.2) | -0.43 (-2.12; 1.26) | -0.24 |
|  | Post | *n*  Baseline  Follow-up | *75*  8.7 (2.0)  9.5 (2.4) | *73*  7.8 (1.9)  8.5 (2.2) | 0.49 (-0.17; 1.14) | 0.24 |
|  | Digit Sequences II | |  |  |  |  |
|  | Pre- and peri- | *n*  Baseline  Follow-up | *9*  6.9 (2.4)  8.7 (3.6) | *11*  6.7 (2.1)  8.0 (2.2) | 0.74 (-2.12; 3.60) | 0.34 |
|  | Post | *n*  Baseline  Follow-up | *75*  6.4 (2.7)  7.5 (2.9) | *73*  5.9 (1.9)  6.8 (2.4) | 0.37 (-0.33; 1.08) | 0.16 |
| **Processing speed** | Reaction Time | |  |  |  |  |
|  | Pre- and peri- | *n*  Baseline  Follow-up | *7*  349 (30)  300 (37) | *10*  388 (62)  327 (42) | -14.9 (-62.3; 32.4) | 0.28 |
|  | Post | *n*  Baseline  Follow-up | *66*  381 (61)  336 (63) | *68*  369 (65)  342 (68) | -12.2 (-31.1; 6.7) | 0.19 |
|  | Connecting the Dots I | |  |  |  |  |
|  | Pre- and peri- | *n*  Baseline  Follow-up | *9*  33.3 (8.5)  29.9 (4.5) | *10*  36.1 (8.3)  31.8 (5.7) | -1.74 (-5.99; 2.51) | 0.21 |
|  | Post | *n*  Baseline  Follow-up | 70  41.1 (9.8)  36.8 (8.2) | 69  38.9 (9.5)  36.0 (9.1) | -0.24 (-2.52; 2.04) | 0.02 |
| **Executive functioning** | Connecting the Dots II | |  |  |  |  |
|  | Pre- and peri- | *n*  Baseline  Follow-up | *9*  54.7 (9.2)  46.1 (6.7) | *9*  55.9 (10.8)  50.8 (8.8) | -3.73 (-10.26; 2.80) | 0.38 |
|  | Post | *n*  Baseline  Follow-up | *68*  69.5 (17.4)  62.8 (18.5) | *72*  70.4 (23.4)  64.5 (18.8) | -1.13 (-5.75; 3.48) | 0.05 |
|  | Place the Beads | |  |  |  |  |
|  | Pre- and peri- | *n*  Baseline  Follow-up | *9*  28.4 (20.2)  22.0 (13.8) | *10*  21.6 (13.5)  14.7 (8.1) | 5.36 (-1.74; 12.46) | -0.32 |
|  | Post | *n*  Baseline  Follow-up | *73*  31.8 (16.9)  27.0 (14.7) | *73*  32.0 (19.1)  27.8 (15.8) | -0.80 (-5.26; 3.66) | 0.04 |
| **Motor functioning** | Fill the Grid | |  |  |  |  |
|  | Pre- and peri- | *n*  Baseline  Follow-up | *9*  64.7 (15.3)  64.1 (10.6) | *11*  70.6 (12.9)  59.8 (9.6) | 6.41 (-4.43; 17.25) | -0.46 |
|  | Post | *n*  Baseline  Follow-up | *73*  74.9 (15.3)  68.7 (12.2) | *72*  72.8 (13.9)  70.0 (13.6) | -2.52 (-5.37; 0.34) | 0.17 |
| **Self-reported cognitive functioning** | MDASI-MM Severity | |  |  |  |  |
|  | Pre- and peri- | *n*  Baseline  Follow-up | *9*  5.3 (1.9)  3.3 (2.1) | *11*  5.8 (1.4)  5.7 (1.7) | -2.42 (-4.30; -0.53) | 1.51 |
|  | Post | *n*  Baseline  Follow-up | *75*  6.1 (1.9)  4.9 (2.3) | *75*  5.6 (1.9)  5.2 (1.8) | -0.51 (-1.10; 0.07) | 0.27 |
|  | MDASI-MM Interference | |  |  |  |  |
|  | Pre- and peri- | *n*  Baseline  Follow-up | *9*  3.6 (1.5)  2.4 (1.8) | *11*  4.5 (1.9)  4.2 (2.0) | -1.18 (-2.93; 0.57) | 0.66 |
|  | Post | *n*  Baseline  Follow-up | *75*  3.6 (2.1)  2.7 (2.2) | *75*  3.2 (1.8)  2.8 (2.1) | -0.34 (-0.89; 0.21) | 0.18 |

Abbreviations: HVLT-R, Hopkins Verbal Learning Test-Revised; MDASI-MM, MD Anderson Symptom Inventory for multiple myeloma.

Values indicate mean (SD).

† The intervention effect is the regression coefficient of a linear regression analysis adjusted for baseline, age and endocrine therapy.

‡ Effect Sizes (ES) were calculated by dividing Beta by the pooled SD at baseline, with positive ESs meaning a beneficial effect of the intervention on a specific outcome. ESs <0.2 indicate “no difference”, ESs between 0.2 and 0.5 indicate “small differences”, ESs between 0.5 and 0.8 indicate “medium differences” and ESs ≥ 0.8 indicate “large differences”[1]. An ES of 0.5 or higher was considered clinically relevant [2].

Note: tests/questionnaires for which a higher score indicates worse performance/functioning/more symptoms: Reaction Time, Connecting the Dots (I & II), Place the Beads, Fill the Grid and MDASI-MM (Severity and Interference).

**Table S6.** Intervention effects on cognitive functioning, stratified for low versus high levels of fatigue measured with the EORTC QLQ C-30 fatigue scale.

| **Cognitive domain** | **Outcome measures** | | **Intervention** | **Control** | **Treatment effect† (95% CI)** | **Effect Size‡** |
| --- | --- | --- | --- | --- | --- | --- |
| **Learning and memory** | HVLT-R total recall | |  |  |  |  |
|  | Low fatigue (<39) | *n*  Baseline  Follow-up | *44*  26.4 (4.6)  26.3 (4.5) | *52*  26.9 (4.2)  26.1 (4.9) | 0.36 (-1.17; 1.88) | 0.08 |
|  | High fatigue (≥39) | *n*  Baseline  Follow-up | *38*  26.8 (4.1)  25.4 (4.2) | *30*  26.5 (4.4)  25.3 (4.6) | 0.09 (-1.76; 1.93) | 0.02 |
|  | HVLT-R delayed recall | |  |  |  |  |
|  | Low fatigue (<39) | *n*  Baseline  Follow-up | *44*  8.8 (2.4)  8.8 (2.6) | *51*  9.4 (2.3)  9.5 (2.3) | -0.42 (-1.19; 0.35) | -0.18 |
|  | High fatigue (≥39) | *n*  Baseline  Follow-up | *38*  9.1 (1.9)  8.9 (2.2) | *30*  9.2 (2.0)  8.8 (2.5) | 0.24 (-0.66; 1.13) | 0.12 |
|  | HVLT-R recognition | |  |  |  |  |
|  | Low fatigue (<39) | *n*  Baseline  Follow-up | *44*  10.8 (1.5)  10.7 (1.5) | *52*  10.8 (1.3)  10.9 (1.5) | -0.15 (-0.68; 0.38) | -0.11 |
|  | High fatigue (≥39) | *n*  Baseline  Follow-up | *38*  10.6 (1.6)  10.6 (1.2) | *30*  10.5 (1.5)  10.3 (1.8) | 0.42 (-0.30; 1.14) | 0.28 |
|  | Wordlist Learning | |  |  |  |  |
|  | Low fatigue (<39) | *n*  Baseline  Follow-up | *44*  42.5 (9.4)  48.8 (8.5) | *53*  44.2 (9.8)  50.4 (9.8) | -0.43 (-2.94; 2.07) | -0.04 |
|  | High fatigue (≥39) | *n*  Baseline  Follow-up | *40*  41.0 (9.8)  49.0 (9.3) | *30*  40.9 (9.6)  45.1 (11.6) | 4.36 (0.47; 8.25) | 0.45 |
|  | Wordlist Delayed Recall | |  |  |  |  |
|  | Low fatigue (<39) | *n*  Baseline  Follow-up | *42*  8.8 (2.2)  10.7 (2.3) | *51*  9.6 (2.6)  11.5 (2.5) | -0.29 (-1.06; 0.49) | -0.12 |
|  | High fatigue (≥39) | *n*  Baseline  Follow-up | *34*  8.1 (3.0)  10.2 (3.0) | *29*  8.2 (2.5)  9.8 (3.3) | 0.74 (-0.68; 2.16) | 0.27 |
|  | Wordlist Recognition | |  |  |  |  |
|  | Low fatigue (<39) | *n*  Baseline  Follow-up | *42*  43.5 (1.6)  44.1 (1.3) | *52*  43.4 (2.0)  43.7 (2.5) | 0.33 (-0.44; 1.10) | 0.18 |
|  | High fatigue (≥39) | *n*  Baseline  Follow-up | *37*  42.3 (3.4)  43.5 (1.8) | *30*  41.9 (3.8)  42.6 (2.9) | 0.96 (-0.06; 1.98) | 0.27 |
| **Attention and working memory** | Box Tapping | |  |  |  |  |
|  | Low fatigue (<39) | *n*  Baseline  Follow-up | *40*  8.9 (1.7)  8.2 (2.3) | *49*  8.6 (2.0)  9.2 (2.1) | -1.21 (-2.11; -0.32) | -0.64 |
|  | High fatigue (≥39) | *n*  Baseline  Follow-up | *38*  8.3 (2.1)  9.2 (1.4) | *29*  8.6 (2.3)  9.5 (1.8) | 0.02 (-0.54; 0.58) | 0.01 |
|  | Digit Sequences I | |  |  |  |  |
|  | Low fatigue (<39) | *n*  Baseline  Follow-up | *44*  8.8 (2.2)  9.6 (2.6) | *53*  8.0 (2.0)  8.4 (2.3) | 0.60 (-0.27; 1.47) | 0.28 |
|  | High fatigue (≥39) | *n*  Baseline  Follow-up | *40*  8.7 (1.9)  9.5 (2.3) | *31*  7.6 (1.5)  9.0 (1.9) | -0.28 (-1.22; 0.65) | -0.16 |
|  | Digit Sequences II | |  |  |  |  |
|  | Low fatigue (<39) | *n*  Baseline  Follow-up | *44*  6.7 (2.6)  7.8 (3.2) | *53*  6.2 (2.2)  7.0 (2.5) | 0.41 (-0.49; 1.31) | 0.17 |
|  | High fatigue (≥39) | *n*  Baseline  Follow-up | *40*  6.3 (2.7)  7.4 (2.8) | *31*  5.8 (1.5)  6.8 (2.2) | 0.34 (-0.79; 1.46) | 0.15 |
| **Processing speed** | Reaction Time | |  |  |  |  |
|  | Low fatigue (<39) | *n*  Baseline  Follow-up | *40*  366 (53)  329 (56.5) | *50*  376 (69)  338 (65.5) | -5.1 (-28.1; 17.8) | 0.08 |
|  | High fatigue (≥39) | *n*  Baseline  Follow-up | *33*  392 (65)  338 (67) | *28*  364 (55)  342 (66) | -26.7 (-52.9; -0.6) | 0.43 |
|  | Connecting the Dots I | |  |  |  |  |
|  | Low fatigue (<39) | *n*  Baseline  Follow-up | *41*  39.7 (10.6)  36.3 (9.0) | *50*  38.8 (9.0)  36.4 (8.5) | -0.40 (-3.20; 2.40) | 0.04 |
|  | High fatigue (≥39) | *n*  Baseline  Follow-up | *38*  40.7 (9.2)  35.8 (7.2) | *29*  38.2 (10.0)  34.0 (9.4) | 0.23 (-3.03; 3.48) | -0.02 |
| **Executive functioning** | Connecting the Dots II | |  |  |  |  |
|  | Low fatigue (<39) | *n*  Baseline  Follow-up | *41*  68.0 (17.5)  62.6 (18.3) | *53*  70.7 (24.2)  63.7 (18.9) | 0.77 (-4.76; 6.31) | -0.04 |
|  | High fatigue (≥39) | *n*  Baseline  Follow-up | *36*  67.5 (17.3)  58.9 (18.3) | *28*  65.2 (19.8)  61.7 (17.7) | -5.06 (-11.89; 1.77) | 0.28 |
|  | Place the Beads | |  |  |  |  |
|  | Low fatigue (<39) | *n*  Baseline  Follow-up | *44*  31.9 (16.9)  25.4 (13.5) | *53*  30.1 (18.0)  24.9 (14.2) | -0.13 (-4.74; 4.49) | 0.01 |
|  | High fatigue (≥39) | *n*  Baseline  Follow-up | *38*  30.9 (17.8)  27.6 (15.9) | *30*  31.8 (20.4)  28.7 (18.0) | -1.34 (-9.11; 6.42) | 0.07 |
| **Motor functioning** | Fill the Grid | |  |  |  |  |
|  | Low fatigue (<39) | *n*  Baseline  Follow-up | *42*  74.3 (17.6)  68.3 (13.2) | *52*  71.9 (15.1)  68.6 (13.3) | -1.32 (-4.89; 2.25) | 0.08 |
|  | High fatigue (≥39) | *n*  Baseline  Follow-up | *40*  73.2 (13.1)  68.1 (11.0) | *31*  73.6 (11.2)  68.7 (14.1) | -2.07 (-6.84; 2.69) | 0.17 |
| **Self-reported cognitive functioning** | MDASI-MM Severity | |  |  |  |  |
|  | Low fatigue (<39) | *n*  Baseline  Follow-up | *44*  5.5 (2.2)  4.3 (2.2) | *54*  5.1 (1.7)  4.8 (1.8) | -0.67 (-1.41; 0.07) | 0.35 |
|  | High fatigue (≥39) | *n*  Baseline  Follow-up | *40*  6.5 (1.5)  5.3 (2.2) | *32*  6.4 (1.7)  6.1 (1.5) | -0.68 (-1.56; 0.21) | 0.43 |
|  | MDASI-MM Interference | |  |  |  |  |
|  | Low fatigue (<39) | *n*  Baseline  Follow-up | *44*  3.1 (2.2)  1.9 (1.8) | *54*  2.7 (1.5)  2.3 (1.9) | -0.67 (-1.31; -0.04) | 0.37 |
|  | High fatigue (≥39) | *n*  Baseline  Follow-up | *40*  4.1 (1.6)  3.5 (2.1) | *32*  4.6 (1.8)  4.1 (2.1) | -0.16 (-1.03; 0.72) | 0.09 |

Abbreviations: HVLT-R, Hopkins Verbal Learning Test-Revised; MDASI-MM, MD Anderson Symptom Inventory for multiple myeloma.

Values indicate mean (SD).

† The intervention effect is the regression coefficient of a linear regression analysis adjusted for baseline, age and endocrine therapy.

‡ Effect Sizes (ES) were calculated by dividing Beta by the pooled SD at baseline, with positive ESs meaning a beneficial effect of the intervention on a specific outcome. ESs <0.2 indicate “no difference”, ESs between 0.2 and 0.5 indicate “small differences”, ESs between 0.5 and 0.8 indicate “medium differences” and ESs ≥ 0.8 indicate “large differences” [1]. An ES of 0.5 or higher was considered clinically relevant [2].

Note: tests/questionnaires for which a higher score indicates worse performance/functioning/more symptoms: Reaction Time, Connecting the Dots (I & II), Place the Beads, Fill the Grid and MDASI-MM (Severity and Interference).

**Table S7.** Intervention effects on patient-reported outcomes.

| **Domain** | **Outcome measures** | **Intervention** | **Control** | **Treatment effect† (95% CI)** | **Effect Size‡** |
| --- | --- | --- | --- | --- | --- |
| **Fatigue** | MFI General fatigue |  |  |  |  |
|  | *n*  Baseline  Follow-up | *84*  15.9 (3.6)  12.4 (4.7) | *84*  15.1 (4.3)  14.0 (5.0) | -2.22 (-3.32; -1.11) | 0.56 |
|  | MFI Physical fatigue |  |  |  |  |
|  | *n*  Baseline  Follow-up | *84*  14.2 (4.2)  9.7 (4.7) | *84*  13.6 (4.5)  12.5 (5.0) | -3.27 (-4.38; -2.15) | 0.76 |
|  | MFI Mental fatigue |  |  |  |  |
|  | *n*  Baseline  Follow-up | *84*  16.4 (3.2)  13.3 (4.1) | *84*  16.5 (3.1)  14.4 (4.1) | -0.98 (-1.95; 0.00) | 0.31 |
|  | MFI Reduced motivation |  |  |  |  |
|  | *n*  Baseline  Follow-up | *84*  11.0 (3.9)  8.3 (3.7) | *84*  10.7 (4.1)  9.2 (3.9) | -1.07 (-1.96; -0.18) | 0.27 |
|  | MFI Reduced activity |  |  |  |  |
|  | *n*  Baseline  Follow-up | *84*  11.8 (4.1)  8.5 (4.3) | *84*  11.5 (4.5)  10.5 (4.7) | -2.11 (-3.15;-1.08) | 0.49 |
| **Quality of Life** | EORTC Summary score |  |  |  |  |
|  | *n*  Baseline  Follow-up | *84*  76.4 (12.8)  80.5 (13.5) | *86*  79.3 (10.9)  78.8 (12.7) | 3.96 (1.21; 6.71) | 0.33 |
|  | EORTC Global health status |  |  |  |  |
|  | *n*  Baseline  Follow-up | *84*  67.0 (17.0)  74.7 (16.1) | *86*  72.6 (14.8)  71.6 (19.7) | 5.82 (1.09; 10.57) | 0.36 |
|  | EORTC Physical functioning |  |  |  |  |
|  | *n*  Baseline  Follow-up | *84*  81.8 (14.0)  85.8 (14.8) | *86*  83.0 (14.0)  84.8 (13.8) | 1.87 (-1.10; 4.83) | 0.13 |
|  | EORTC Cognitive functioning |  |  |  |  |
|  | *n*  Baseline  Follow-up | *84*  49.6 (22.8)  61.7 (23.8) | *86*  51.4 (19.3)  57.9 (23.1) | 4.99 (-0.37; 10.35) | 0.24 |
|  | EORTC Role functioning |  |  |  |  |
|  | *n*  Baseline  Follow-up | *84*  65.7 (25.3)  73.2 (25.9) | *86*  73.6 (24.0)  70.7 (23.0) | 7.17 (1.27; 13.06) | 0.29 |
|  | EORTC Emotional functioning |  |  |  |  |
|  | *n*  Baseline  Follow-up | *84*  71.9 (21.8)  77.5 (21.6) | *86*  77.3 (17.9)  79.7 (16.8) | 1.27 (-3.18; 5.72) | 0.06 |
|  | EORTC Social functioning |  |  |  |  |
|  | *n*  Baseline  Follow-up | *84*  71.0 (28.9)  81.3 (23.9) | *86*  78.9 (22.8)  79.7 (22.3) | 5.92 (0.22; 11.62) | 0.23 |
|  | EORTC Fatigue |  |  |  |  |
|  | *n*  Baseline  Follow-up | *84*  42.6 (23.3)  36.6 (23.1) | *86*  35.4 (19.8)  37.0 (22.1) | -4.49 (-10.08; 1.10) | 0.21 |
|  | EORTC Pain |  |  |  |  |
|  | *n*  Baseline  Follow-up | *84*  25.6 (27.9)  26.6 (26.1) | *86*  20.0 (21.7)  25.2 (25.7) | -2.19 (-8.33; 3.94) | 0.08 |
|  | EORTC Insomnia |  |  |  |  |
|  | *n*  Baseline  Follow-up | *84*  38.5 (32.9)  38.5 (32.5) | *86*  36.0 (29.5)  34.9 (31.5) | 2.03 (-5.87; 9.93) | -0.07 |
| **Depression** | PHQ-9 |  |  |  |  |
|  | *n*  Baseline  Follow-up | *84*  7.7 (5.1)  5.6 (4.9) | *86*  6.4 (3.8)  5.9 (4.4) | -1.16 (-2.19; -0.13) | 0.26 |
|  | HADS Depression |  |  |  |  |
|  | *n*  Baseline  Follow-up | *84*  6.5 (3.8)  4.6 (3.6) | *84*  6.1 (3.6)  4.8 (3.4) | -0.46 (-1.23; 0.31) | 0.12 |
| **Anxiety** | HADS Anxiety |  |  |  |  |
|  | *n*  Baseline  Follow-up | *84*  8.2 (3.6)  6.8 (4.1) | *84*  7.9 (3.8)  6.3 (4.3) | 0.23 (-0.62; 1.07) | -0.06 |

Abbreviations: MFI, Multidimensional Fatigue Inventory; EORTC, European Organisation for Research and Treatment of Cancer Quality of Life Questionnaire; PHQ-9, Patiënt Health Questionnaire-9; HADS, Hospital Anxiety and Depression Scale.

Values indicate mean (SD).

† The intervention effect is the regression coefficient of a linear regression analysis adjusted for baseline, age and endocrine therapy.

‡ Effect Sizes (ES) were calculated by dividing Beta by the pooled SD at baseline, with positive ESs meaning a beneficial effect of the intervention on a specific outcome. ESs <0.2 indicate “no difference”, ESs between 0.2 and 0.5 indicate “small differences”, ESs between 0.5 and 0.8 indicate “medium differences” and ESs ≥ 0.8 indicate “large differences” [1]. An ES of 0.5 or higher was considered clinically relevant [2].

Note: questionnaires for which a higher score indicates more symptoms: MFI subscales, EORTC Fatigue, EORTC Pain, EORTC Insomnia, HADS Anxiety & Depression, PHQ-9.

**References**

1. Cohen J. Statistical Power Analysis for the Behavioral Sciences, Academic press, 2013.

2. Norman GR, Sloan JA, Wyrwich KW. Interpretation of changes in health-related quality of life the remarkable universality of half a standard deviation. Med. Care 2003; 41(5):582–592.
